# Secular change of true polar wander over the past billion years

**DOI:** 10.1126/sciadv.abo2753

**Published:** 2022-10-14

**Authors:** Hairuo Fu, Shihong Zhang, Daniel J. Condon, Hanbiao Xian

**Affiliations:** ^1^ State Key Laboratory of Biogeology and Environmental Geology, China University of Geosciences, Beijing 100083, China.; ^2^ Department of Earth and Planetary Sciences, Harvard University, Cambridge, MA 02138, USA.; ^3^ NERC Isotope Geosciences Laboratory, British Geological Survey, Keyworth NG12 5GG, UK.

## Abstract

The rate of movement of Earth’s solid shell relative to its spin axis, or true polar wander, depends on variations in mantle convection and viscosity. We report paleomagnetic and geochronologic data from South China that constrain the rate of rapid true polar wander (>5° per million years) between 832 million years and 821 million years ago. Analysis of the paleomagnetic database demonstrates secular change of true polar wander related to mantle cooling and thermal structure across supercontinent cycles. True polar wander rates are relatively muted with a partially insulated mantle during supercontinent assembly and accelerate as mantle thermal mixing reestablishes with supercontinent breakup. Decreasing true polar wander rate through the Neoproterozoic was succeeded by overall smaller variations in the Phanerozoic. We propose that Neoproterozoic extensive plate tectonic activities enhanced mantle cooling, giving rise to a reduction in mantle convective forcing, an increase in mantle viscosity, and a decrease in true polar wander rates into the Phanerozoic.

## Teaser

Mantle cooling and thermal structure control secular change of true polar wander rates.

## INTRODUCTION

True polar wander (TPW) is the rotation of a planet or moon’s entire solid exterior relative to its spin axis in response to changes in its moment of inertia associated with mass redistribution (*1*). Two first-order controls have been proposed to dictate Earth’s TPW rate in geological time: the magnitude of internal inertia perturbations, particularly convective loading that scales with the vigor of mantle convection, and the viscosity of the lower mantle, which is temperature dependent (*2*–*5*). These two properties coevolve over Earth’s history and are modulated by the secular cooling of the mantle, through which decreasing mantle temperature leads to less vigorous convection and higher viscosity, which should limit rapid TPW (*2*–*5*).

Paleomagnetic data constrain TPW to ≤3° million year^−1^ (Ma^−1^) during the Phanerozoic (ca. 539 Ma ago to present) (*6*–*9*). More rapid TPW (>4° Ma^−1^) has been revealed mostly from Neoproterozoic rock records (1000 to 539 Ma ago) (*10*–*15*). For example, the hypothesized ca. 810 to 795 Ma ago Bitter Springs TPW based on paleomagnetic results from the Akademikerbreen Group in Svalbard implied ~83° of cumulative continental motion in ~15 Ma (*10*). However, without direct dates on paleomagnetic poles, uncertainty has lingered in the inferred TPW rates (*10*–*12*). Similarly, the significance of ca. 850 to 800 Ma ago pole motions of Baltica (~90°) remains unresolved without more precise age constraints (*16*, *17*). Moreover, discerning rapid TPW events depends on the geocentric axial dipole (GAD) hypothesis, and large Ediacaran pole shifts have been alternatively speculated to reflect the component of non-GAD fields (*18*, *19*). Last, determining the pure TPW signal involves removing simultaneous tectonic movements from the composite pole shift that comprises the two, which has been a longstanding issue to concern and circumvent (*20*).

To estimate TPW rates from the pole shifts complicated by tectonic movements, which maintain largely uncertain in the Neoproterozoic, new paleomagnetic poles with records of uniformitarian geomagnetic fields and tight geochronologic controls are required. With precise age determinations of a pole pair, tectonic movements during the time interval can be modeled and subtracted to quantify the pure TPW composition. We have acquired high-precision U-Pb chemical abrasion thermal ionization mass spectrometry (CA-TIMS) zircon dates coupled with paleomagnetic poles from Neoproterozoic mafic sills, which intruded in the lower Fanjingshan Group, Guizhou Province, South China. Integrated with existing global data, our new results found an exceptionally rapid TPW event (>5° Ma^−1^ based on the best estimation) between 832 and 821 Ma ago, leading to further analysis of the global paleomagnetic database to explore the mechanisms for the changing TPW rate in Earth’s history. Our analysis suggests a geodynamic linkage that relates the observed overall decrease and periodic fluctuations in TPW rates with mantle cooling and alternating thermal structures across supercontinent cycles. The resolved geodynamic coupling provides new arrays of predictions for Earth’s thermal history and implications for the coevolution of Earth’s interior systems and rotational stability.

## RESULTS

The Fanjingshan mafic sills experienced subgreenschist-grade metamorphism and were folded in the Jiangnan Orogeny (ca. 830 to 815 Ma) (*21*, *22*), permitting regional fold tests for the paleomagnetic results. We sampled oriented paleomagnetic cores from a total of 12 diabase sills in five sections, with intruded host-rock samples from the Jinzhanping section for a baked contact test (fig. S1). The intrusive contacts of the sampled sills are parallel to bedding (figs. S1 and S2). Five zircon crystals were selected from a geochronologic sample, TS01C, collected from a gabbro-diabase sill for CA-TIMS analysis (fig. S1).

### Geochronology

Zircon grains selected for CA-TIMS analysis were euhedral, elongated, similar-sized (~200 to 300 μm) and, with sharp terminations, interpreted to represent crystallization from the same magma. Isotope and age results are reported in table S1. ^206^Pb/^238^U versus ^207^Pb/^235^U ratios show concordant and coherent results (fig. S3A), indicating successful removal of potential lead loss in zircon grains after annealing and chemical abrasion procedures. The weighted mean ^206^Pb/^238^U date calculated from the zircon analyses gives 831.51 ± 0.32 Ma (2σ uncertainty) (fig. S3 and table S1), representing the crystallization age of the mafic sill.

### Paleomagnetism

Seven sills entailing 125 specimens yielded stable and consistent high-temperature components. Two low-temperature components, LT1 and LT2, were first removed during thermal demagnetization. LT1, separated mostly below ~450°C, is present in most samples and identified as a viscous remanent magnetization of the recent geomagnetic field (figs. S5 and S6). LT2 (300° to 350°C), a southeast-up direction in geographic coordinates, is only found from the five sills in the Huguosi and Jingzhanping sections (figs. S5 and S6). LT2 fails a fold test and is also secondary (fig. S6), but its age and origin are relatively unconstrained.

A stable high-temperature component, HT1, carried by magnetite, is identified from all seven sills (~500° to 580°C) and their baked contacts (~480° to 550°C) (fig. S5). HT1 passes the McFadden fold test (*23*) at 95% confidence level and a progressive unfolding test (*24*) that shows the tightest distribution obtained at 98.9% unfolding (fig. S7), suggesting a magnetization acquired before the folding of the Fanjingshan Group by ca. 815 Ma ago (*12*). Paleomagnetic results from the intruded host rocks in the Jinzhanping section demonstrate a positive baked contact test: Baked siltstones record a similar HT1 from that of the sills, while unbaked siltstones preserve a distinct high-temperature component (HT2) from HT1 and other directions determined in the region (fig. S9). The positive baked contact test and fold tests indicate that HT1 is a primary magnetization acquired during the cooling of the sills at 831.51 ± 0.32 Ma.

**Fig. 1.**
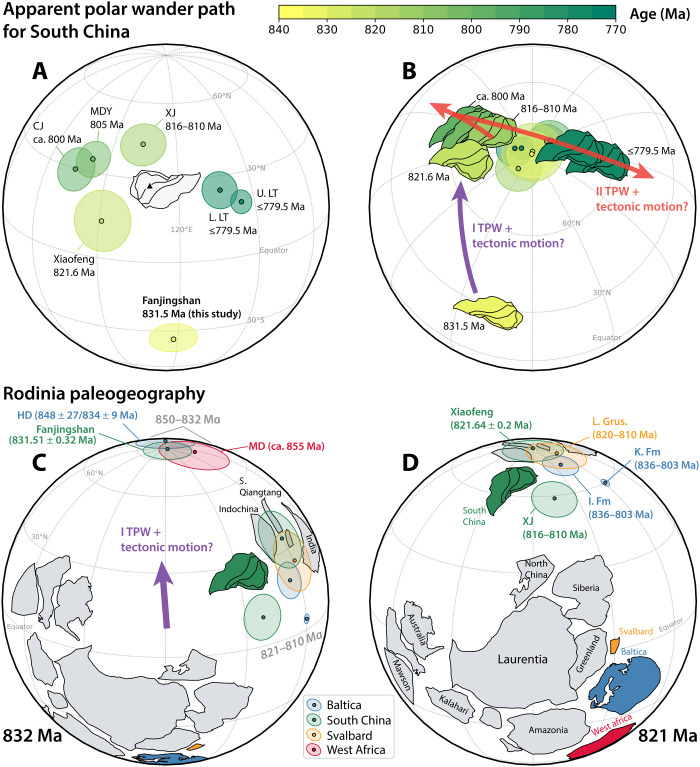
Mid-Tonian paleogeographic models for South China and Rodinia. (**A**) Tonian apparent polar wander path (APWP) of South China. Ellipses show *A*_95_, the 95% confidence cones of the poles. The triangle signifies the mean sampling locality of the Fanjingshan sills (27.92°N and 108.69°E) for paleolatitude conversion. See table S3 for details of the data. (**B**) The proposed paleogeographic model for South China reconstructed with the APWP in (A). Paleogeographic changes between 816 and 780 Ma ago follow the preferred “minimize difference” model from (*12*). Hybrid TPW + tectonic motions are interpreted for 832 to 821 Ma ago and 816 to 795 Ma ago. (**C** and **D**) Rodinia paleogeographic changes in the context of 832 to 821 Ma ago “type I” TPW. Poles and Euler rotation parameters are provided in tables S3, S4, and S7. CJ, Chengjiang Formation pole; MDY, Madiyi Formation pole; XJ, Xiajiang Group pole; L. LT, lower Liantuo Formation pole; U. LT, upper Liantuo Formation pole; HD, Hunnedalen dykes pole; MD, Manso dykes pole; L. Grus., lower Grusdievbreen Formation pole; K. Fm, Katav Formation pole; I. Fm, Inzer Formation pole.

The corresponding paleomagnetic pole of HT1 (the Fanjingshan pole; 34.7°S and 118.2°E; *A*_95_ = 8.6) does not resemble any younger pole reported from South China, further supporting a primary origin (Fig. 1A and fig. S11). Multiple dispersion parameters of virtual geomagnetic poles (VGPs) fall within expected values of paleosecular variation models (fig. S11) (*25*, *26*), supporting that the Fanjingshan pole has sufficiently recorded the time-averaged position of the geomagnetic pole at ca. 832 Ma ago, during which the fidelity of the GAD field is also independently substantiated by magnetostratigraphic studies of coeval sedimentary successions (*17*) and comprehensive analyses of the Precambrian geomagnetic field [e.g., (*27*)].

## DISCUSSION

### Discovery of rapid TPW between 832 and 821 Ma ago

The new Fanjingshan pole places the sampling location at a paleolatitude of ~27° at ca. 832 Ma ago (Fig. 1A). The Fanjingshan pole and the 821.64 ± 0.2 Ma ago Xiaofeng dykes pole (*28*, *29*) quantify the paleolatitudinal displacement of South China to ~41° between 832 and 821 Ma ago, at an average rate of ≥46.5 ± 15.7 cm year^−1^ (Fig. 1A). Observational and theoretical speed limits of modern plates are ~19 and ~20 cm year^−1^, respectively (*30*, *31*). The mean latitudinal plate velocity implied by the Fanjingshan and Xiaofeng poles is at least twice the upper limits of the known plate tectonic speeds. It is thus interpreted to record a combined signal of tectonic movements and TPW. The total pole shift between 832 and 821 Ma ago is quantified to be 54.7° ± 13.9° with a mean rate of 5.5° ± 1.4° Ma^−1^.

**Fig. 2.**
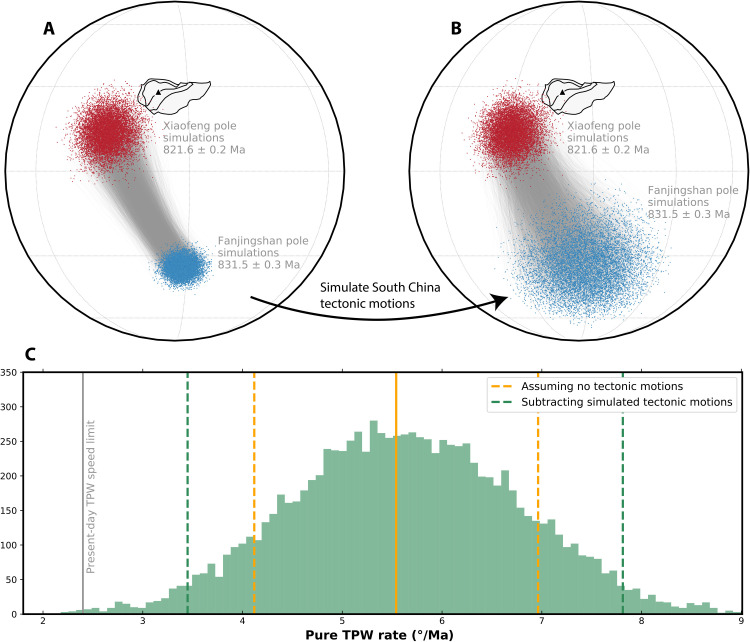
TPW rate (832 to 821 Ma ago) estimated using Monte Carlo analysis. (**A**) Resampled Fanjingshan and Xiaofeng poles from Fisher distribution (*n* = 10,000 for each). Pole pairs are randomly drawn between the two pole groups, connected with fine gray lines representing angular distance. The triangle indicates the mean sampling locality of the Fanjingshan sills (27.92°N and 108.69°E). (**B**) Simulation of South China tectonic motions between 832 and 821 Ma ago assuming a speed limit of implied fastest Neoproterozoic plate tectonic velocities (~31 cm year^−1^) (*56*) with unconstrained directions and application of these displacements to the resampled Fanjingshan poles from (A). The resultant new pole pairs reflect the pure TPW component after subtracting simulated tectonic motions. Dividing the pure TPW angular distances by the age differences between the pole pairs yields pure TPW rate estimates. (**C**) Histogram of pure TPW rate estimates from (B). The green dashed lines bound the 95% confidence intervals of the TPW rate after accounting for simulated tectonic motions (3.5° to 7.8° Ma^−1^). The orange solid and dashed lines show the mean rate and 2σ uncertainties of the total pole shift between the Fanjingshan and Xiaofeng poles using SE propagation (5.5° ± 1.4° Ma^−1^) (see Materials and Method), which could reflect TPW estimates only when assuming no tectonic motions of South China. The gray solid line shows the proposed TPW speed limit (~2.4° Ma^−1^) set by present-day mantle viscosity estimates (*2*). See Materials and Methods for details.

South China was either disconnected entirely from Rodinia (*12*, *32*, *33*) or located at its periphery during the Tonian (1000 to 720 Ma ago) (*34*). The direction and speed of the tectonic motions of South China between 832 and 821 Ma ago are currently unknown, because of a paucity of exactly coeval data from other continents for comparison of relative tectonic motion. Considering that signals of TPW and tectonic motion might enhance or counteract each other (*20*), implementing a Monte Carlo method, we simulate tectonic motions of South China between 832 and 821 Ma ago, by which the total pole shifts are corrected to constrain quantitatively the pure TPW component (Fig. 2 and fig. S15) (see Materials and Methods). The disentangled 832 to 821 Ma ago TPW rate has an estimated 95% confidence interval of 3.5° to 7.8° Ma^−1^ (Fig. 2C). The rate is comparable to the hypothesized Bitter Springs TPW constrained to be ≥3.6° to 4.9° Ma^−1^ for the entire Bitter Springs stage (from ca. 810 to <795 Ma ago) (*10*, *11*) and ≥5.4° Ma^−1^ for the first portion of the TPW oscillation (ca. 810 to 795 Ma ago) (*12*).

**Fig. 3.**
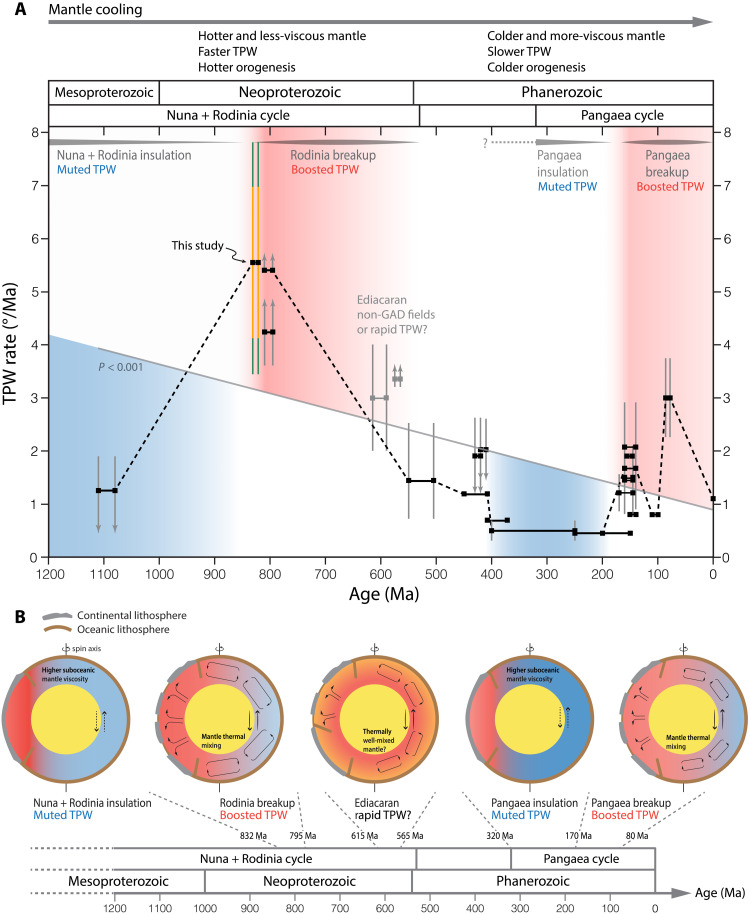
Secular change of the TPW rate and our proposed geodynamic coupling. (**A**) Probable TPW rate estimates since the late Mesoproterozoic. TPW rates and uncertainties are summarized in Supplementary Text and tabulated in table S8. The gray solid line indicates a linear regression fit of the data (*P* < 0.001) that characterizes the first-order decreasing trend (the fit excludes the Ediacaran data of debated origins, shown as gray squares). See fig. S14 for sensitivity tests that also consider variously interpreted Ediacaran TPW rate estimates. The black dashed line traces the second-order variation in TPW connected by representative rate estimates of individual time intervals. Gray vertical bars show the uncertainties of rate estimates. Up/down arrows indicate that the rate could be above/below the shown range defined by the error bars. For the TPW rate uncertainty of this study, the orange bar represents considering no tectonic motions and the green bar reflects accounting for simulated tectonic motions (corresponding to those shown in Fig. 2C). (**B**) Schematic illustration of the secular change of TPW related to mantle cooling and alternating mantle thermal structures linked to assembly and breakup of continents (supercontinent aggregation–mantle thermal insulation–muted TPW; supercontinent breakup–mantle thermal mixing–boosted TPW).

Large paleolatitudinal continental motions during the Tonian were also revealed from the paleomagnetic poles of Baltica (*16*, *17*, *35*, *36*). These poles, dated roughly between 850 and 800 Ma ago, demonstrate a total rotation of ~90°. The pole comparison is illustrated in Fig. 1 (C and D), which shows the lining up of the apparent polar wander paths (APWPs) of South China and Baltica between ca. 850 and 810 Ma ago, reconstructed in relative configurations consistent with those proposed for Rodinia (for Euler rotation parameters and references, see table S7). This ca. 850 to 810 Ma ago Rodinia APWP is further consolidated by the ca. 850 Ma ago Manso dykes pole from West Africa (*37*) and the ca. 820 to 810 Ma ago lower Grusdievbreen Formation pole from East Svalbard (*10*), which both fall closely along the APWP defined by the South China and Baltica poles (Fig. 1, C and D). Both East Svalbard and Baltica are considered part of assembled Rodinia in the Tonian (*10*, *36*). The discovery of comparable prominent paleogeographic shifts of congruent Rodinia and presumably separate South China supports the hypothesis that these movements record an extended period of rapid TPW from 850 to 795 Ma ago: 850 to 820 Ma ago equatorward migration of Rodinia and its associated geoid high (“type I” TPW) (Fig. 1, C and D) followed by 810 to 795 Ma ago oscillatory motions about the center of Rodinia (“type II” TPW) once the positive geoid docked and stabilized in the equatorial zone (*3*, *5*, *20*, *38*, *39*). Similar to Rodinia, Pangaea was proposed to have witnessed a similar fashion of TPW succession from northward migration (400 to 250 Ma ago, type I TPW) to equator-centered oscillations (250 to 100 Ma ago, type II TPW) while at lower average rates (Fig. 3A) (*3*, *39*).

### Secular change of TPW over the past billion years

The findings of 850 to 795 Ma ago rapid TPW events enable contextualizing the Tonian observations in the evolution of TPW over time. The inferred high Tonian TPW rates exceed the speed limit of observed TPW through the Phanerozoic (~3° Ma^−1^) (*6*–*9*) and theoretical constraints (~2.4° Ma^−1^) (*2*) with present-day lower mantle viscosity estimates. Numerical simulations suggest that rapid TPW at >4° Ma^−1^ with a maximum rate of ~6° Ma^−1^ can be achieved with substantially increased convective flow (*4*) and/or reduced lower mantle viscosity to ~3 × 10^21^ to 10 × 10^21^ Pa·s, 10 to 30% of the present value (*3*) consistent with thermal history modeling for the Tonian (*40*). These comparisons support a more vigorously convecting and less-viscous mantle in the Tonian that allowed more rapid reorganization between principal moments of inertia. We synthesize and analyze the extant probable estimates for the TPW rate over the past billion years (Fig. 3A and table S8). The time series manifests a general decreasing nature of the rate since the Tonian, a remarkable drop toward the Cambrian (from 832 to 505 Ma ago), and a relatively more stable state through the Phanerozoic (505 to 0 Ma ago) (Fig. 3A). Two TPW peaks occur at around 832 to 795 Ma ago and 170 to 80 Ma ago, coinciding with the early breakup of Rodinia and Pangaea, respectively (*41*, *42*). The Ediacaran rapid polar wander rates (conservative estimates) appear to be compatible with an implied TPW reduction from the Tonian to the early Phanerozoic if TPW rather than non-GAD fields was the driving mechanism (Fig. 3A) (*13*, *15*).

We propose a geodynamic link to explain both the systematic decrease in the TPW rate and episodic variations imposed over the mean trend since the late Mesoproterozoic, in which mantle cooling coupled with supercontinent cycles. The observed overall secular TPW decline is consistent with TPW rate controlled primarily by increasing lower mantle viscosity and declining vigor of mantle convection as Earth’s interior cooled (Fig. 3A and fig. S14) (*3*–*5*). The two high TPW stages, between 832 to 795 Ma ago and 170 to 80 Ma ago (Fig. 3A), coincide with transitions in the Rodinia and Pangaea supercontinent cycles, preceded by protracted supercontinent thermal insulation and succeeded shortly by supercontinent dispersal (*42*–*44*). During the 1800 to 850 Ma ago Nuna-Rodinia lifetime (*44*, *45*) and the 320 to 180 Ma ago Pangaea lifetime (*7*), supercontinents rimmed by peripheral subduction zones may have partially insulated the underlying mantle and altered mantle thermal structures, warming the subcontinental mantle and cooling the suboceanic mantle (temperature variation of order 100°C or greater) (*46*–*48*). This insulating regime resulted in inhibited convective mixing between the subcontinental and suboceanic domains, localized mantle flow pattern relative to the supercontinent, raised viscosity of voluminous suboceanic mantle, and associated decreased velocity of oceanic plates (*46*, *47*), setting the stage for slow TPW (Fig. 3, A and B). The 1110 to 1080 Ma ago TPW rates appear to have been restrained more considerably, a presumed consequence of longer-lived insulation by Nuna and Rodinia compared to Pangaea, creating greater lateral mantle thermal and viscosity variations (*46*, *47*).

As heat accumulated beneath the subcontinental region and lateral hydrostatic pressure gradient built up in the mantle, the peripheral subduction girdle was destabilized and a supercontinent approached the wake of dispersal (*39*, *46*–*48*). Breakdown of a subduction girdle would reestablish large-scale advective exchange between the subcontinental and suboceanic mantle, relaxing mantle thermal and viscosity anomalies (*46*). This mantle thermal mixing involves unleashing a pulse of increased convective vigor driven by the preexisting lateral temperature gradient, accelerated overturn of oceanic plates, and enhanced global convective motions (*45*–*48*), invigorating mass redistributions in Earth’s interior and on the surface that speed up TPW. Meanwhile, as mantle thermal reequilibrium proceeds, warming the once-cooled and more-viscous suboceanic mantle might reduce the maximum viscosity of the lower mantle (rate-limiting TPW), which potentially relieves the restriction on TPW rates (*5*). Such a changeover of mantle thermal state could take over ~100 Ma to accomplish (*46*–*48*) and may explain the accelerated TPW rates prompted at 832 to 795 Ma ago, 170 to 145 Ma ago, and ca. 80 Ma ago, succeeding the breakup of Rodinia and Pangaea (Fig. 3). Following supercontinent breakup, elevated mantle flow, surface heat flux, and lithospheric mobility would have amplified mantle cooling rates initially, followed by gradual restoration to a baseline-cooling trend as convective forcing weakens toward the completion of mantle thermal mixing (*46*–*48*). The relaxed convective vigor contributes to the subsequent decline and stabilization of TPW rates until the formation of the next supercontinent cycle (Fig. 3A). These findings underscore the increasing importance of accounting for large-scale and varying lateral thermal heterogeneities associated with mantle thermal structures in future numerical modeling of TPW.

Notably, the notable reduction in the TPW rate during the Neoproterozoic might suggest an irreversible transition, after which the mantle cooled enough to render a slow-TPW regime in the Phanerozoic (<3° Ma^−1^), also characterized by an overall smaller amplitude of variation in TPW rates (Fig. 3A). This implied mantle thermal decline in the Neoproterozoic resonates with coeval lithospheric evidence showing a rising dominance of low thermobaric ratios (*T*/*P*) of rock records since the Tonian that subsequently persisted through the Phanerozoic (*44*, *49*). The promoted and continual prevalence of low *T*/*P* metamorphic records, i.e., the blueschist-facies and ultrahigh-pressure metamorphism, is inferred to be directly linked to cooling of the upper mantle, leading to colder, denser, and strengthened oceanic lithosphere and deeper slab breakoff in the collision zones (*49*, *50*). Numerical models suggest that such a transition in the style of orogenesis from hotter to colder may have taken place as the mantle temperature fell to ~80° to 100°C higher than present-day values (*50*), corresponding to an inferred Neoproterozoic timing constrained by igneous rock records and thermal modeling (*51*). This mantle heat loss may have been facilitated by plate tectonic activity since the Tonian associated with the breakup of Rodinia (peaking at ca. 800 to 600 Ma ago) and the assembly of Gondwana (ca. 650 to 500 Ma ago) (*52*). In particular, cooling may have been facilitated by extensive circum-supercontinent subduction zones and internal rifting (*52*). Consequently, by the early Cambrian, progressively reduced mantle heat, which would have led to suppressed mantle convection and raised viscosity, in turn, deactivated mantle physical conditions favorable for prompting rapid TPW in succeeding periods (Fig. 3A).

Characterizing the secular change of TPW raises further opportunities and considerations in paleogeographic reconstructions. The established framework provides testable predictions for facilitating subsequent explorations of TPW to improve and revise our extant understanding. Despite an expanding global TPW database, records of well-defined TPW excursions have remained, generally, elusive. Nevertheless, the importance of high-resolution sampling in resolving fast while smaller-amplitude TPW (~10°) has been demonstrated recently (*9*), which suggests more potential missing TPW signals to search for, especially during the dynamic TPW stages as our model predicts (Fig. 3). With better characterization of TPW from new observations, full accounting of TPW motions, including periods throughout supercontinent transitions, may become available to aid in the solution of absolute paleolongitude of ancient plates (*53*). The secular change of TPW also gives insights into tracing the changing rate of plate tectonics. As TPW manifests with coherent motion of global plates, the intervals of large-scale TPW would reinforce the sensitivity in quantifying plate tectonic rates concurrently with TPW, as already explored by analyzing differences of contemporaneous pole motions [e.g., (*12*, *54*)]. Most existing estimates of Proterozoic plate velocities are not corrected for possible TPW [e.g., (*55*)], particularly for the Neoproterozoic when TPW was likely faster and more variable. Discerning rates between TPW and tectonic movement in Earth’s deep time would enable probing possible coupling of the two in the long term, both functions of evolving mantle thermal history and cyclic transformations in continental configurations and mantle structures (*1*, *3*–*5*, *31*, *46*).

## MATERIALS AND METHODS

### Field sampling

Paleomagnetic cores were collected using a portable gasoline-powered drill with nonmagnetic, diamond-rimmed bites. The intrusive contacts of the sampled mafic sills are parallel to the bedding of the country rocks (sandstone and siltstone of the lower Fanjingshan Group). Sills with observable contacts were prioritized for sampling. The seven sills that yielded stable and consistent high-temperature components used to calculate the pole all have direct bedding controls. Multiple bedding measurements were made for the country rocks closely adjacent to the contacts to calculate the mean bedding for each sill (table S2). The thickness of the sampled sills is typically over 10 m. Each sill was drilled evenly across its thickness to average the characteristic remanent magnetization. Where measured close to the sampling sites, no significant bedding difference was observed for the upper and lower contacts of each sill, indicating a consistent emplacement of each sill along bedding at each of the sampling localities. Therefore, we adopt a common mean bedding for samples collected from the same sill. We measured the orientation of the cores in the field using a combination of the magnetic compass and sun compass when the sun was available. No significant difference is observed between the two orientation methods.

### U-Pb geochronology

Zircon separation from block samples was performed at Harvard University using standard techniques. Zircon U-Pb geochronology was conducted in the Isotope Laboratory at Natural Environment Research Council (NERC) Isotope Geoscience Laboratories (NIGL) at British Geological Survey. Separated zircon crystals were annealed in a muffle furnace at 900°C for 60 hours in quartz beakers. Selected zircon grains were transferred to 3 ml of Hex Savillex beakers. They were processed through ultrasonic bathing and rinsing with 30% HNO_3_ and then moved to 300 μl of Teflon perfluoroalkoxy alkane (PFA) microcapsules to be leached in an about 5:1 mix of 29 M hydrofluoric acid (HF) and 30% HNO_3_ for 12 hours at 180°C. After removing the leachate, the residues were rinsed again with 30% HNO_3_ and 6 M HCl and spiked with the EARTHTIME ^235^U-^233^U-^205^Pb tracer (*57*). Each zircon grain was then fully dissolved in about 120 μl of 29 M HF with a trace amount of 30% HNO_3_ at 220°C for 48 hours. The solutions were then dried, and fluorites in the samples were converted to chlorides in 3 M HCl at about 180°C overnight. Standard HCl-based anion exchange chromatographic procedures were performed to separate U and Pb from each sample and then loaded on a single Re filament in a silica gel–phosphoric acid mixture. Isotope ratios of U and Pb were analyzed using the Thermo-Electron Triton TIMS system in NIGL. U isotopes were measured in static Faraday mode or on a single scanning electron microscopy (SEM) detector, depending on the uranium contents. Pb isotopes were measured using the peak-hopping method with a single SEM detector. Data reductions were processed in Tripoli (*58*). U-Pb concordia diagrams were plotted using IsoplotR (*59*).

### Paleomagnetic and rock magnetic experiments

Paleomagnetic and rock magnetic experiments were performed in Paleomagnetism and Environmental Magnetism Laboratory at the China University of Geosciences, Beijing. Oriented paleomagnetic cores were first sliced into cylindrical specimens with a thickness of ~1 to 2.2 cm using nonmagnetic, diamond-rimmed saw blades. Remanent magnetizations of all specimens were measured with a 2G 755-4 K three-axis cryogenic magnetometer in a magnetic shield room with a <300-nT residual field. Specimens were processed through stepwise thermal demagnetization in either an ASC TD-48 or MMTDSC furnace that holds an internal residual field of <10 nT. Most specimens were completely demagnetized in ~20 to 35 steps by up to ~580°C. Temperature increment between each step varies between 20° and 40°C in the low-temperature range (<500°C) and 3° to 10°C in the high-temperature range (500° to 580°C). Some specimens were subject to small demagnetizing increments of 5° to 10°C between 300° and 350°C to characterize better the removal of low-temperature components in this interval. To identify the carriers of magnetic remanences, the temperature-dependent low-field susceptibility of representative samples (ground to fine powders) was measured in an argon atmosphere with an AGICO KLY-4S Kappabridge with CS-3 high-temperature device. Heating and cooling curves were acquired between room temperature and 700°C.

### Paleomagnetic analyses and calculations

Remanence components were fit using linear principal components analysis (*60*). Unless specified, we force the best-fit lines to pass through the origin when fitting the high-temperature components. Only specimens with ≥4 stable points that decay to the origin with MAD (maximum angle of deviation) smaller than 20° in the high-temperature interval are regarded as credible and used to calculate specimen directions. Specimen directions were then averaged using Fisher statistics (*61*) to calculate the site mean direction of each sill/site of sedimentary rocks. Specimen analysis was done using the Paleomag OS X program (*62*). Paleomagnetic analyses and plotting were completed in Python facilitated by the PmagPy package (*63*).

To quantify the rate of pole shift between a pair of poles, we incorporate full error propagation in the calculation considering uncertainties in both the ages and positions of the poles. Given the great-circle distance [Δθ (in degrees)] and age difference [Δ*t* (in million years)] between two poles, *a* and *b*, the mean rate of pole shift (in degrees per million years) with 2σ error is calculated as follows

$$\text{rate}=\frac{\Delta \theta }{\Delta t}(1\pm \sqrt{\frac{{\sigma_{\left(a\right)}}^{2}+{\sigma_{\left(b\right)}}^{2}}{{\left(\Delta t\right)}^{2}}+\frac{{A_{95(a)}}^{2}+{A_{95(b)}}^{2}}{{\left(\Delta \theta \right)}^{2}}})$$ (1)

where *A*_95_ is the radius of the 95% confidence cone of the pole and σ is the 2σ uncertainty on the pole age.

We take a Monte Carlo approach to further constrain the range of pure TPW rates between 832 and 821 Ma ago inferred from the Fanjingshan and Xiaofeng poles. From Fisher distributions, a large number (10,000) of random VGPs are simulated for each paleomagnetic pole according to their pole positions and associated uncertainties (table S3), paired with random draws from Gaussian distributions for the pole ages (831.51 ± 0.32 Ma and 821.64 ± 0.2 Ma). Simulated VGPs from each pole group are paired randomly, connected by gray arcs shown in Fig. 2A.

To account for the potential pole shift produced by tectonic movement and to disentangle the pure TPW component, we simulate tectonic movements of South China between 832 and 821 Ma ago and subtract them from the total pole motion. The direction and velocity of tectonic movements of South China during this period are currently unclear. Hence, for simulating the tectonic speed, we adopt a triangular distribution (fig. S15) assuming:

1) The lower speed limit at 0 cm year^−1^.
2) The mode at 10 cm year^−1^ that corresponds to the fastest plate velocity of South China extracted from the Neoproterozoic plate kinematic models by (*55*).
3) The upper speed limit at 31 cm year^−1^ that represents the possible fastest tectonic movement implied since the late Mesoproterozoic (*56*).

Unconstrained directions of South China tectonic movements are assumed to accommodate the largest effect of tectonic motions. We take 10,000 random draws from the tectonic speed distribution described above (fig. 15), and directions of the tectonic motions are randomly sampled from 0° to 360° from uniform distributions. These simulated tectonic speeds paired with random directions are multiplied by the simulated age differences to obtain the distances of tectonic movements between 832 and 821 Ma ago. The series of tectonic movements are then randomly applied to the sampled Fanjingshan poles in Fig. 2A to convert the effect of plate motions to its paleomagnetic expression. Figure 2B shows the displaced Fanjingshan poles after correcting for simulated tectonic motions, leaving the new angular difference between each pole pair an estimate of the pure TPW component. Last, the pure TPW angular distance is divided by the age difference between the pole pair to estimate the pure TPW rate (Fig. 2C). The 2.5 and 97.5 percentiles are taken from the rate distribution, interpreted as the 95% confidence intervals (Fig. 2C). Accounting for tectonic motions yields a larger spectrum of TPW rate estimations compared with assuming zero plate displacement (Fig. 2C). The prolonged upper tail corresponds to scenarios where the total pole motion is produced by TPW counteracted by tectonic motion, resembling the case interpreted for South China between 815 and 800 Ma ago (*12*) and North China in the late Jurassic (*54*), whereas the reduced lower tail corresponds to TPW and tectonic motion moving along similar directions and acting additively, similar to the interpretation for Laurentia between ca. 1110 and 1080 Ma ago (*56*).

## Supplementary Materials

### This PDF file includes:

Supplementary Text Figs. S1 to S15 Tables S1 to S8 References
