# Supplementary material for: Secular change of true polar wander over the past billion years
Source: Sci Adv. 2022 Oct 14;8(41):eabo2753. doi: 10.1126/sciadv.abo2753 (PMC9565807; doi:10.1126/sciadv.abo2753)
Supplement: Supplementary file 1 — Supplementary Text Figs. S1 to S15 Tables S1 to S8 References [file sciadv.abo2753_sm.pdf]

Science Advances
AAAS

Supplementary Materials for

Secular change of true polar wander over the past billion years

Hairuo Fu *et al.*

Corresponding author: Shihong Zhang, shzhang@cugb.edu.cn

Sci. Adv. **8**, eabo2753 (2022)
DOI: 10.1126/sciadv.abo2753

The PDF file includes:

Supplementary Text
Figs. S1 to S15
Tables S1 to S8
Legend for data file S1
References

Other Supplementary Materials for this manuscript includes the following:

Data file S1

Supplementary Text

Geologic setting of the Fanjingshan Group

The Fanjingshan area, northeastern Guizhou province, South China (fig. S1A), lies in the southwest segment of the ca. 830–815 Ma Jiangnan Orogen, which united the Yangtze and Cathaysia blocks to form the South China craton (*12*, *22*). The 870-815 Ma Fanjingshan Group (Gp) are the oldest exposed rocks in the region and consist predominantly of clastic sedimentary rocks with mafic volcanic rocks and intrusions (ca. 830 Ma sills) (*21*, *22*, *64*). The tectonic setting of the Fanjingshan Gp has been interpreted a retro-arc basin along the southwestern continental margin of the overriding Yangtze during the Yangtze-Cathaysia convergence, with sedimentary grains sourced from both Yangtze block and the juvenile continental magmatic arc (*22*, *64, 65*). The Fanjingshan Gp and its mafic sills underwent greenschist-facies to sub-greenschist-facies metamorphism and were folded, uplifted, and eroded in the Jiangnan Orogeny (*21*). The Fanjingshan Gp is unconformably overlain by the ca. 815–717 Ma Xiajiang Gp, a succession of clastic sedimentary rocks with interbedded volcanic ashes (*12*, *66*) (fig. S1B). The regional deformation of the Fanjingshan Gp sealed by this unconformity is constrained to have occurred between ca. 830 and 815 Ma and has been interpreted as a manifestation of the final collision between the Yangtze and Cathaysia blocks (*12, 22*).

The Fanjingshan Gp is divided into lithologically distinctive lower and the upper parts. The lower Fanjingshan Gp, which includes the Taojinhe, Yujiagou, Xiaojiahe, and Huixiangping formations, is ~3500–6800 m thick and is composed of fine-grained clastic sedimentary rocks and mafic volcanic rocks (including pillow basalts) and sills (*21*, *64*, *66*). The upper Fanjingshan Gp, which includes the conformable Tongchang, Waxi, and Duyantang formations, is a ~3500 m thick succession of mostly coarse-grained sedimentary rocks (interpreted as turbidites) and minor felsic tuffs (*18*, *64*, *66*). The mafic sills occur only in the lower Fanjingshan Gp and are absent in the upper Fanjingshan Gp (*18*, *64*, *66*). These sills and the Fanjingshan Gp were folded along NE-SW axis (fig. S1B) (*64*, *67*), indicating deformation of the Fanjingshan Gp postdates the sill intrusions. The typical thickness of the sills is from meters to tens of meters.

Previous geochronologic investigations have constrained the deformation of the Fanjingshan Gp to be after ~830 Ma, determined by U-Pb laser ablation inductively coupled plasma mass spectrometry (LA-ICP-MS) zircon dates on sills from the lower Fanjingshan Gp [827 ± 15 Ma and 830.8 ± 4.4 Ma (*21*); 831 ± 6 Ma and 827 ± 24 Ma (*68*)] and tuffs from the upper Fanjingshan Gp [832.0 ± 8.5 Ma (*69*)]. The U-Pb CA-ID-TIMS zircon date on the gabbro-diabase sill from this study constrains deformation to be <831.51 ± 0.32 Ma. The maximum depositional age of the Fanjingshan Gp is estimated to be ca. 870–840 Ma based on U-Pb LA-ICP-MS dates on detrital zircon (*64*) and tuffs [840 ± 5 Ma and 851.3 ± 4.0 Ma (*69*, *70*)] in the lower Fanjingshan Gp.

The interpretation that the Fanjingshan Gp formed in a retro-arc basin is supported by (i) coexisting input of detrital grains from old continental (Archean to Mesoproterozoic) and juvenile arc (ca. 880–820 Ma) sources (*22*, *64*, *65*, *71*); (ii) predominantly continental geochemical affinities of the detrital zircon (*65*); and (iii) detrital zircon age spectrum closely resembling those from inner Yangtze and characteristically distinguished from those from Cathaysia (*71*). Radiogenic isotopic composition of the subduction-related igneous rocks from the Fanjingshan Gp [high initial $^{87}Sr/^{86}Sr$ and mostly negative εNd(t)] is consistent with contribution of continental basement to the

magma (*22*). The large proportion of old continental-derived detritus (~45%) in the Fanjingshan Gp suggests deposition occurred proximal to the inferred Yangtze source region (*22*, *65*). The inferred retro-arc setting of the Fanjingshan Gp and its equivalents in the southwest Jiangnan Orogen contrast to the intraoceanic arc settings proposed for the northeast Jiangnan domain (Shuangxiwu and Qigong groups), which lack old continental-derived grains and instead display a unimodal early Neoproterozoic age population (970–850 Ma), and preserve juvenile, depleted mantle isotopic signatures in the igneous suites with minimal apparent crustal contamination (*22*, *65*).

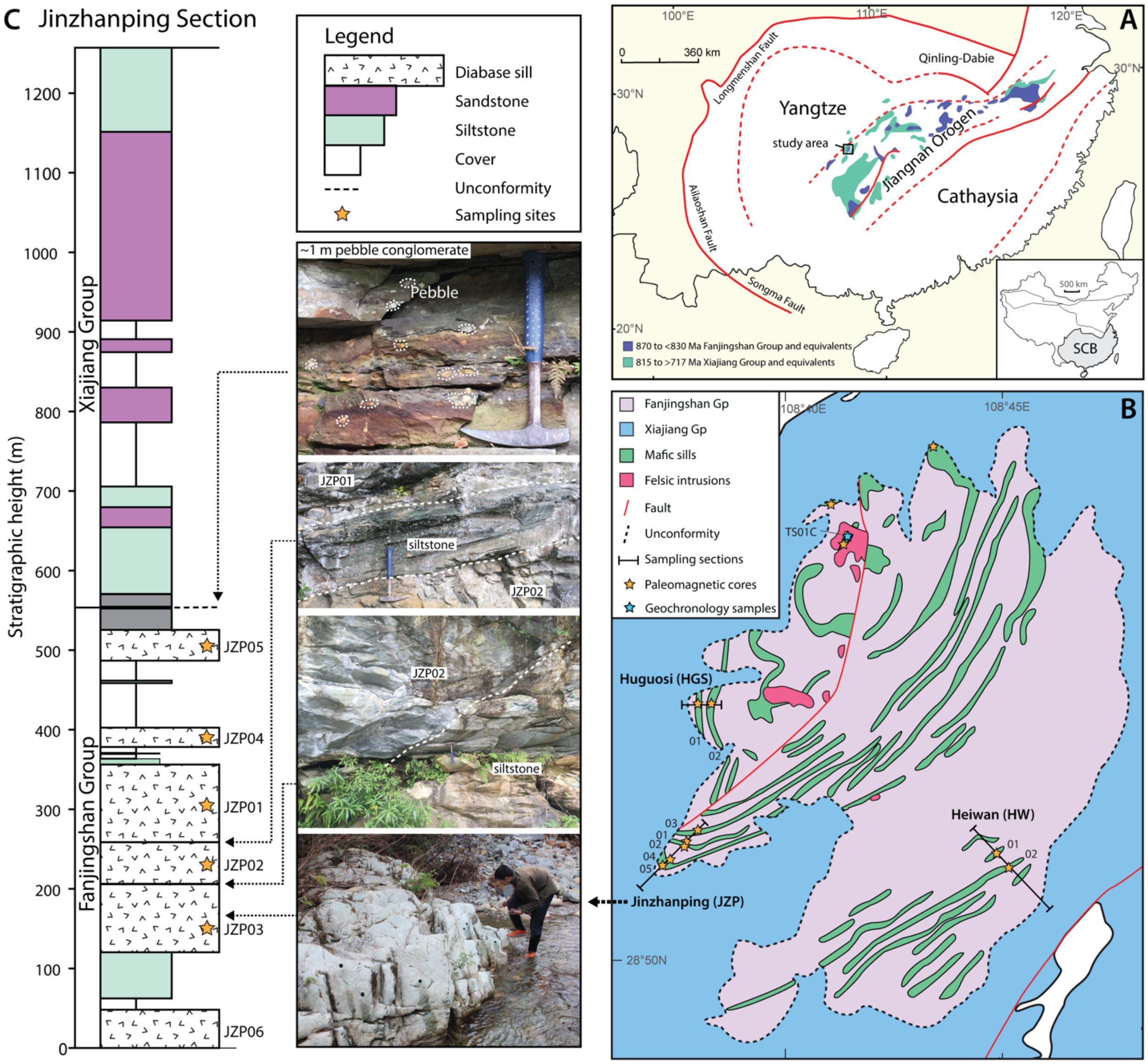

Fig. S1. Geological setting of the Fanjingshan area and measured stratigraphic sections. (**A**) Locations of the Tonian Fanjingshan Group and its equivalents in South China [modified from (*34*)]. (**B**) Simplified geological map of the Fanjingshan area. Stars indicate sampling localities of paleomagnetic and geochronological samples. (**C**) Measured stratigraphy of the Jingzhanping (JZP) section [modified from (*12*)]. Sampling sites are where paleomagnetic cores were collected. The unconformity between the Fanjingshan Group and the Banxi Group in the JZP section is marked by a ~1 m pebble conglomerate and inconsistent bedding across this boundary. Colors of the stratigraphic columns reflect the color of sedimentary rocks. Intrusive contacts of the diabase sills and country rocks are contoured with white dashed lines in photos.

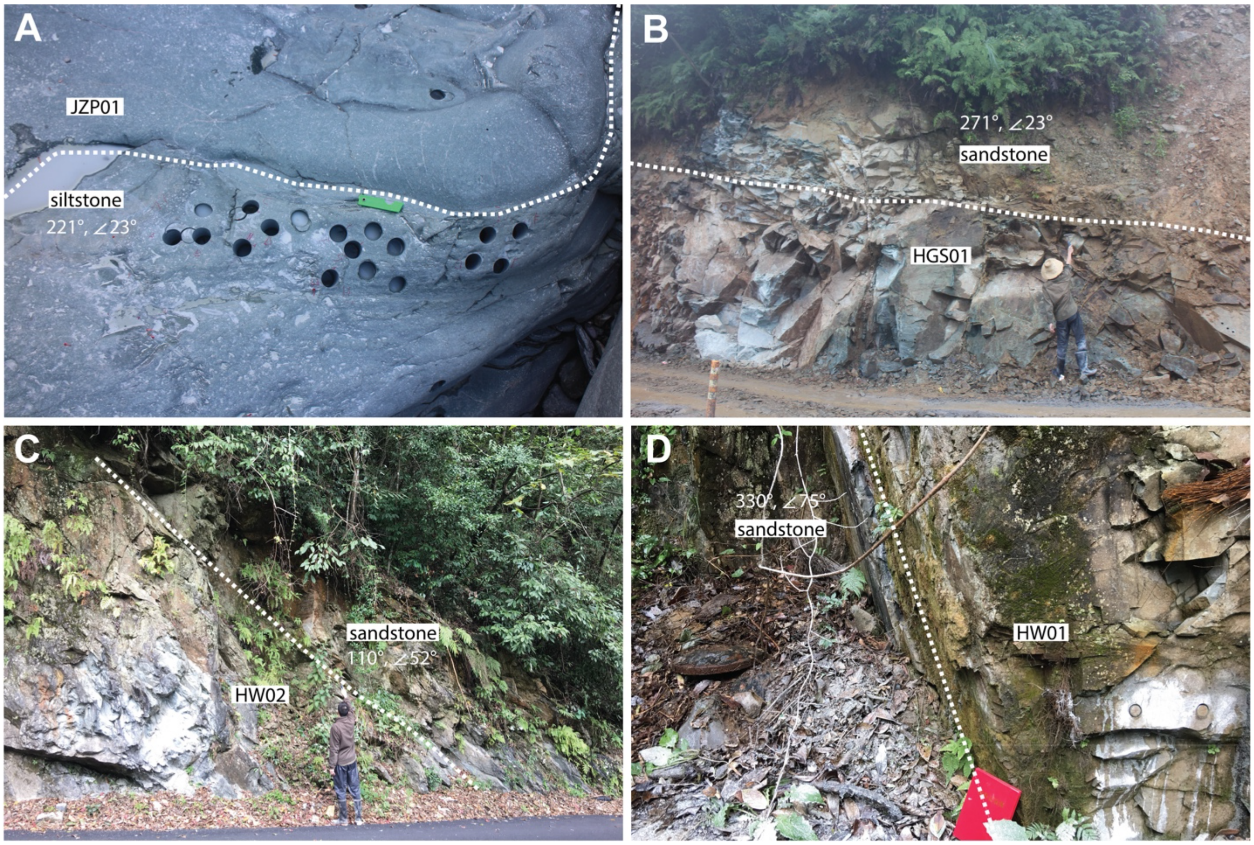

Fig. S2. Field phots of sampled diabase sills in the Fanjingshan Group. See fig. S1C and Table S2 for the locations of the rock units. (A) shows the intrusive contact of JZP01 and sampling sites of the baked contact.

Additional description of geochronologic results

A medium-grained gabbro-diabase sample (TS01C), composed primarily of plagioclase and pyroxene, was selected for dating. $^{206}Pb/^{238}U$ vs. $^{207}Pb/^{235}U$ ratios for the analyzed five zircons of TS01C are plotted on the concordia curve (fig. S3A). Filled ellipses and vertical bars are zircon grains included in the age calculation (fig. S3). The weighted mean $^{206}Pb/^{238}U$ age calculated by the four zircon analyses is 831.51 ± 0.32 Ma, giving the crystallization age of the diabase sample.

Rock magnetic results

Thermal magnetic susceptibility experiments for representative samples reveal a significant decrease in the susceptibility around 580°C, suggesting magnetite is the major magnetic carrier of the samples from the sills and country rocks (fig. S4A–G). An increase in susceptibility after ~425°C in samples and the cooling curves not resembling the heating curves indicate magnetic mineralogy changes after heating experiments. Thermal demagnetization data show no abnormal change in direction or magnitude of the remanence in the high-temperature range (fig. S5), showing that the potential alterations of magnetic grains do not influence the remanent magnetization. Fractional magnetization vs. temperature diagram (fig. S4H) manifests two substantial intensity losses between 500–580°C and 300–350°C. The former corresponds to the unblocking temperature range of fine-grained (titano) magnetite; the latter overlaps with the typical unblocking temperature of pyrrhotite at ~320°C and is only observed for samples from the HGS and JZP sections. The 500–580°C magnetization drop appears to be less remarkable for samples from the JZP section, a result of relatively lower intensity held in the high temperature range compared to the total magnetization (fig. S4H). Together, these results suggest that magnetite is the main magnetic carrier of the remanence. Pyrrhotite is inferred to be present in part of the samples that accounts for the sudden intensity decline at ~320°C.

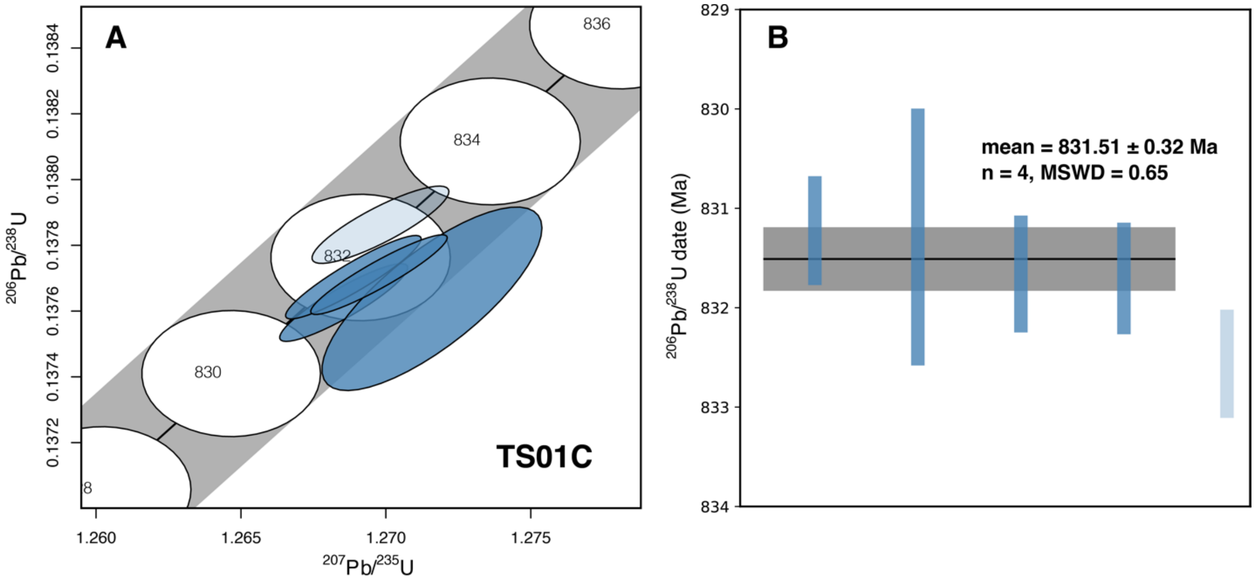

Fig. S3. U-Pb CA-ID-TIMS results for the diabase sample TS01C. (**A**) U-Pb Concordia plots for the analyzed diabase sample, TS01C. Ellipses represent 2σ uncertainty of individua zircon analyses. The light grey band shows analytical uncertainty associated with the decay constants and $^{238}U/^{235}U$ ratio. (**B**) Weighted-mean plot of the $^{206}Pb/^{238}U$ ages. Vertical bars reflect 2σ uncertainty of individual zircon analyses. The black horizontal line and grey band signify the weighed mean date and its 2σ uncertainty (internal precision) calculated for the sample. Filled ellipses and vertical bars are zircons included in age calculation. MSWD—mean squared weighted deviation.

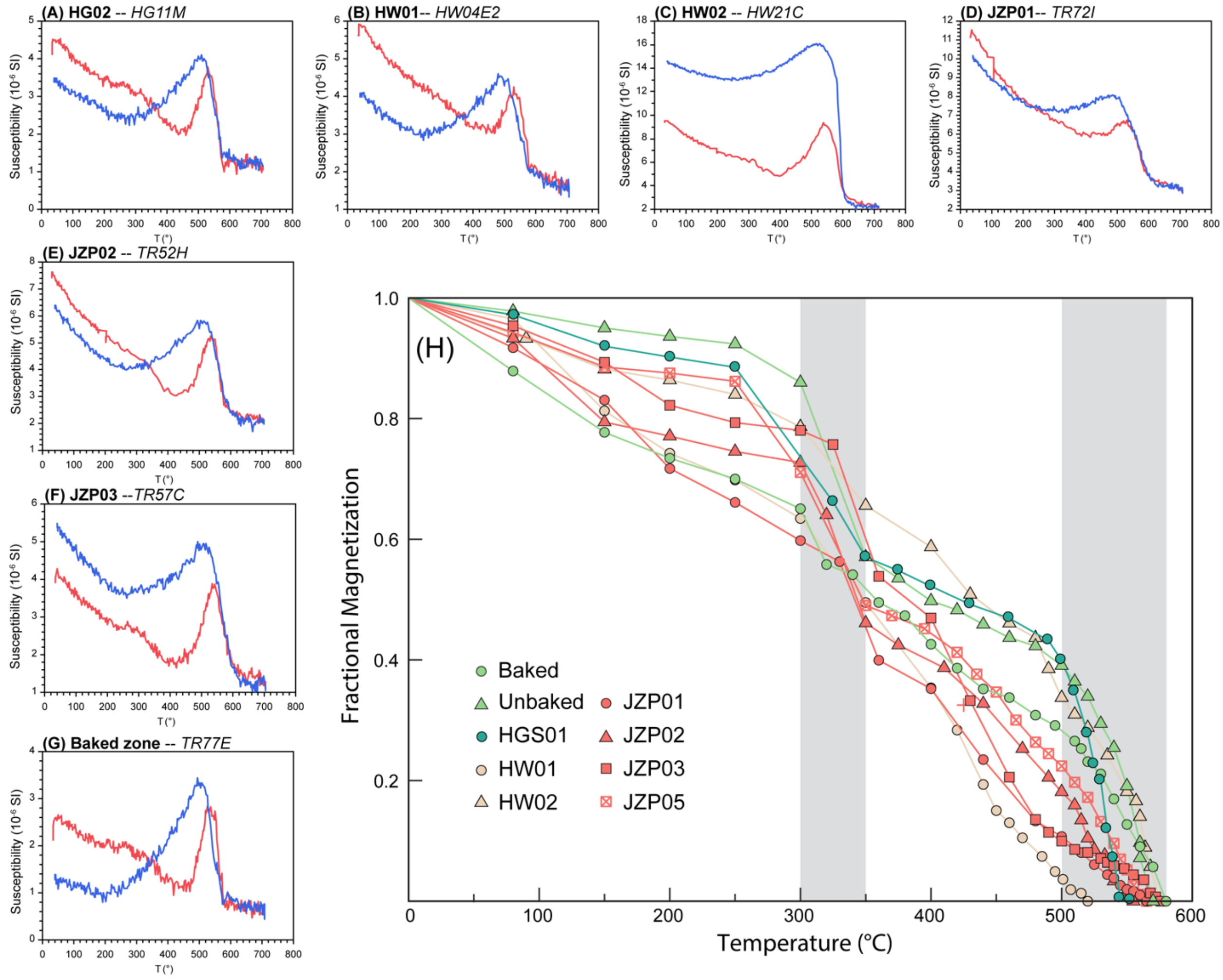

Fig. S4. Rock magnetic analyses of samples from the Fanjingshan Group. (**A–G**) Magnetic susceptibility vs. Temperature (*K*-*T*) results for representative paleomagnetic samples from each rock unit. Lines in red are heating curves and in blue are cooling curves. The title of each diagram signifies the rock-unit ID followed by sample ID. Locations and lithologies of the rock units are provided in Table S2. (**H**) Fractional magnetization vs. thermal demagnetization for representative samples. Between two demagnetization steps, the intensity loss is calculated as the magnitude of the demagnetized vector, instead of the arithmetic difference between the remanent magnetizations. Baked—baked country rocks of the sill JZP01. Unbaked—unbaked country rocks in the JZP section. Vertical grey bands highlight the temperature interval of 300–350°C and 500–580°C.

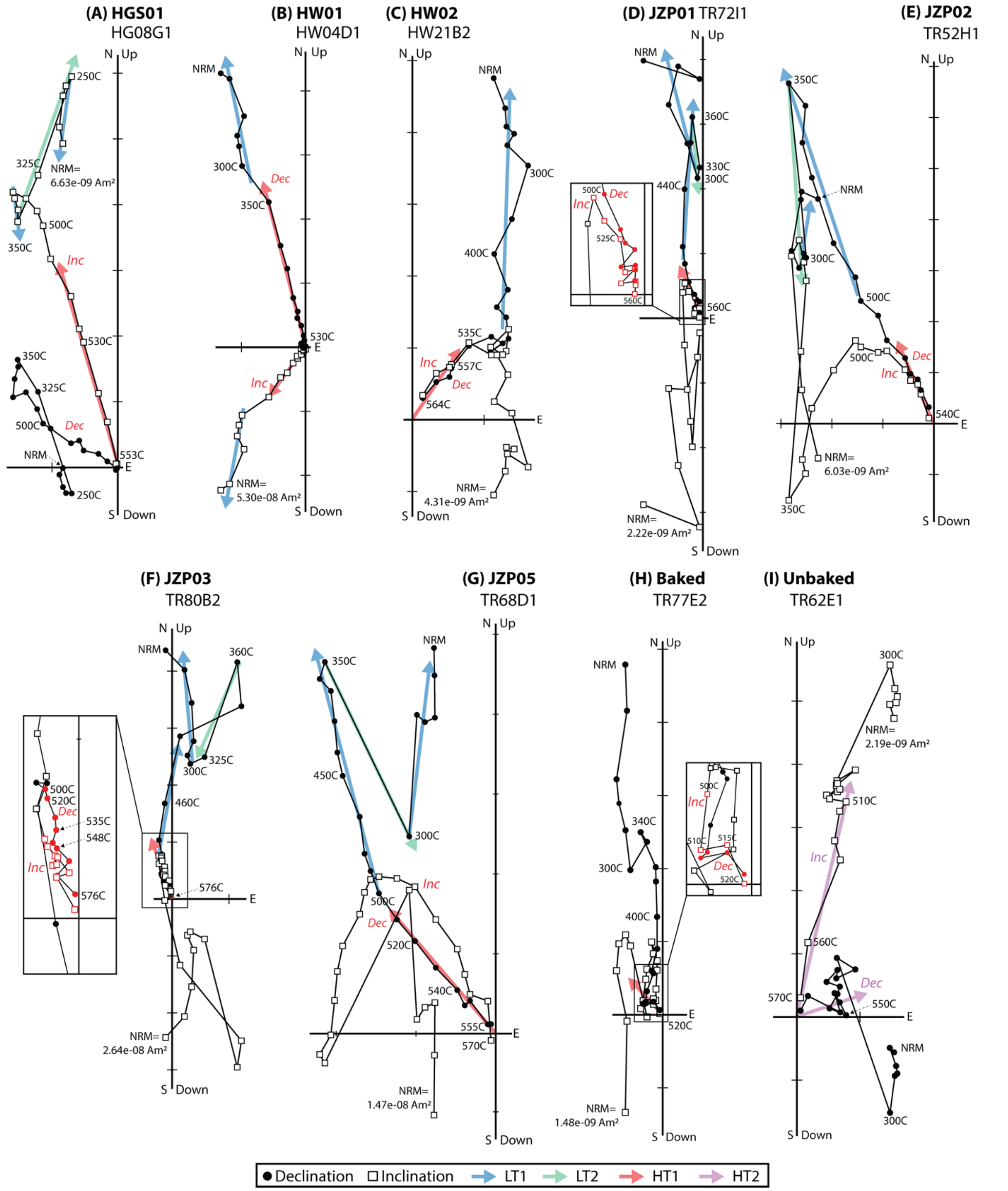

Fig. S5. Vector component diagrams of representative thermal demagnetization data. Directions are shown in geographic coordinates. Each title signifies the name of the rock unit and the specimen (Table S2). Vectors in colors indicate the least-squares fits for each remanence component (*60*). NRM—natural remanent magnetization; LT1 and LT2—low-temperature components; HT1—high-temperature component carried by sills and baked rocks; HT2—high-temperature component carried by unbaked rocks in the JZP section.

Low temperature components (LT1 and LT2)
Most of the analyzed samples, including sills and country rocks, carry LT1. LT1 was typically removed from room temperature to ~450°C during thermal demagnetization, unless intermitted by the removal of MT between ~300–350°C for part of the samples (fig. S5). The mean of LT calculated from all samples in geographic coordinates is D = 357.5°, I = 47.3°, n = 193, α_{95} = 1.9°, close to the local present field direction and fails a fold test (fig. S6). These suggest LT1 is a viscous remanent magnetization (VRM) of the local present geomagnetic field.

LT2 resides in samples from the Huguosi (HGS) and Jingzhanping (JZP) sections but not from the Heiwan (HW) section. LT2 was isolated from a relatively narrow range, between ~300–350°C, entailing the typical unblocking temperature of pyrrhotite (~320°C) (fig. S5). LT2 has variable magnetization that accounts for about 10–40% of the samples' total natural remanent magnetization (NRM) (fig. S4H). The mean of LT2 of all samples in geographic coordinates is D = 171.8°, I = -43.9°, n = 135, k = 47.7, α_{95} = 1.8° (fig. S6). In the vector component diagrams where LT2 is present, the removal of LT2 between ~300–350°C displays a visible directional shift, showing an opposite trend to the LT1 removal after which LT1 resumes decay. LT2 also fails a fold test and is interpreted as secondary (fig. S6). However, the age and origin are unclear. Both unstable components, LT1 and LT2, are not further evaluated in the study.

High-temperature component (HT1) and positive regional fold tests
We average the specimen directions of each sill to calculate the site-mean directions of HT1 of the seven sills. In geographic coordinates, HT1 display scattered distributions that fall into three groups—a NW-up direction of sills from the HGS and JZP sections, a NW-down direction of HW01, and a NE-up direction of HW02 (fig. S7). After tilt-correction using the beddings of each sill's country rocks (sedimentary rocks of the lower Fanjingshan Gp), HT1 became significantly clustered (D = 351.3°, I = -44.1°, N = 7, k = 46.5, α_{95} = 8.9°) (fig. S7). The seven site-mean directions pass the McFadden (*23*) fold test at the 95% confidence level [critical ξ = 3.1; ξ_1 (*in-situ*) = 3.8; ξ_1 (*tilt-corrected*) = 1.4]. A progressive unfolding test (*24*) indicates that the highest precision parameter (k) is obtained at 98.9% unfolding (fig. S7), enveloped by 89–110% suggested for an optimal positive fold test (*73*). Together, the positive regional fold tests suggest HT1 was acquired before the tilting and folding of the Fanjingshan Gp when the sills were parallel to country rocks of the lower Fanjingshan Gp.

We also compute the HT1 mean using all the specimen directions (specimen mean) to test the stability of HT1 directions calculated by different statistical approaches. The specimen mean of HT1 in tilt-corrected coordinates, D = 350.3°, I = -44.9°, n = 125, k = 20.4, α_{95} = 2.9° (fig. S8), closely resembles the mean calculated by the seven site-mean directions of the sills (D = 351.3°, I = -44.1°, N = 7, k = 46.5, α_{95} = 8.9°) (fig. S7). Compared with the specimen mean, the mean of the site-mean directions is a better representative of HT1 because, in this way, it avoids the potential bias toward the groups of specimen directions with larger sample sizes.

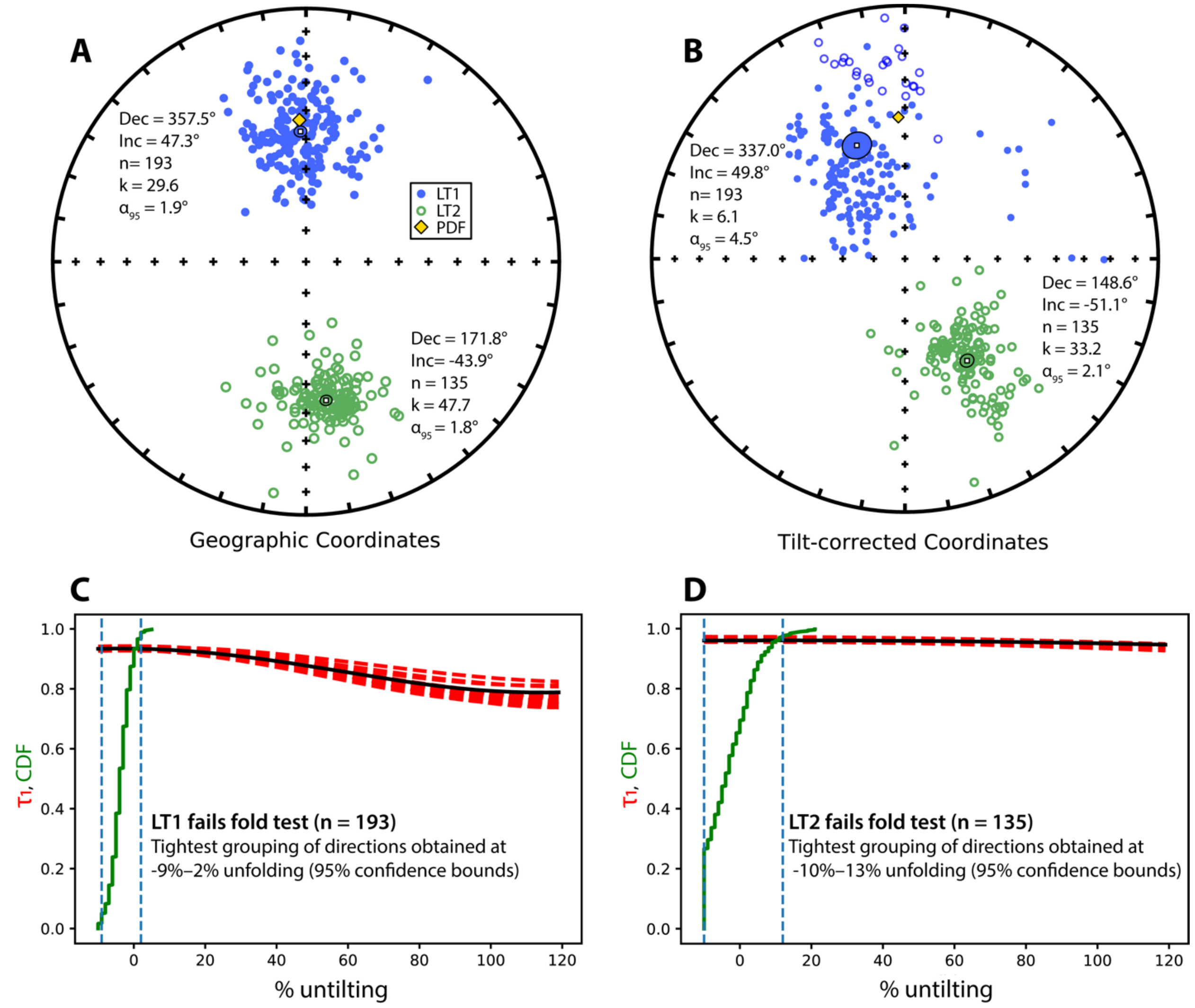

Fig. S6. Specimen directions of LT1 and LT2 and fold tests. (**A**) and (**B**) Equal-area plots of LT1 and LT2 of all the specimens from which they are found. Filled circles indicate downward directions, and open circles indicate upward directions. Ellipses show the 95% confidence intervals of the means (α_{95}). PDF—the present-day field direction of the mean sampling area after International Geomagnetic Reference Field (IGRF) data. Dec—declination; Inc—inclination; k—precision parameter; n—number of samples for statistics. **(C)** and (**D**) Results for the Tauxe and Watson (*72*) fold test on LT and MT. τ1, maximum eigenvalue shown as the black solid line, is the criteria for estimating the unfolding degree given data distributions in geographic coordinates. Red dashed curves are the results of the 500 times bootstrap simulations. Blue dashed lines bound the 95% confidence intervals of the best estimated unfolding. CDF—cumulative distribution function.

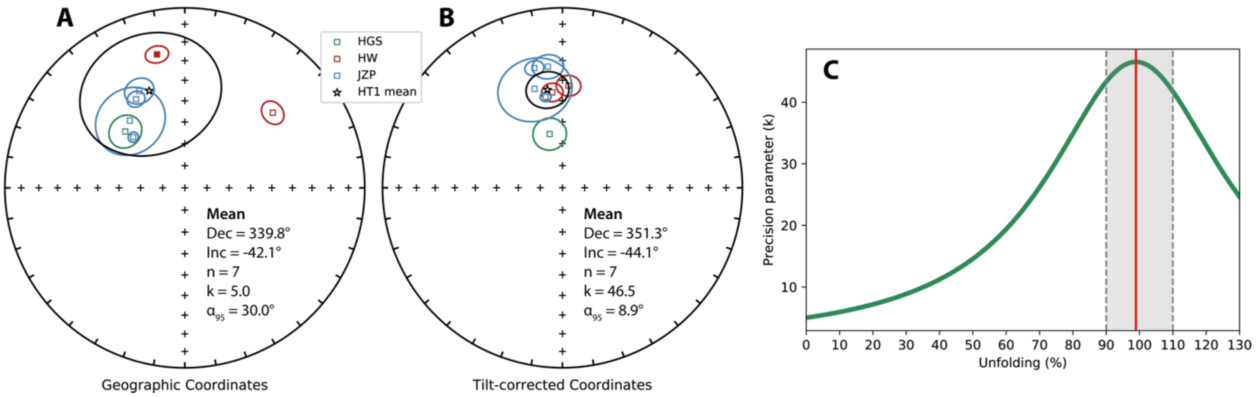

Fig. S7. Site-mean directions of HT1 of the seven sills. (**A**) & (**B**) Equal-area plots of HT1 from all the seven sills in geographic and tilt-corrected coordinates (Table S2). Color-filled/color-rimmed squares represent lower/upper hemisphere mean directions; stars indicate the means; ellipses show radius of 95% confidence cone of the means (α_{95}). Stars indicate the means of the seven site-mean directions. Dec—declination; Inc—inclination; k—precision parameter; N—number of directions to calculate the mean; HGS—Huguosi section; HW—Heiwan section; JZP—Jinzhanping section. (**C**) Progressive unfolding test (*24*) for HT1 using the site-mean directions of the seven sills. The red vertical line at 98.9% unfolding indicates where *k* reaches its maximum. The dashed vertical lines bound 90–110% unfolding preferred for a positive fold test suggested by (*73*).

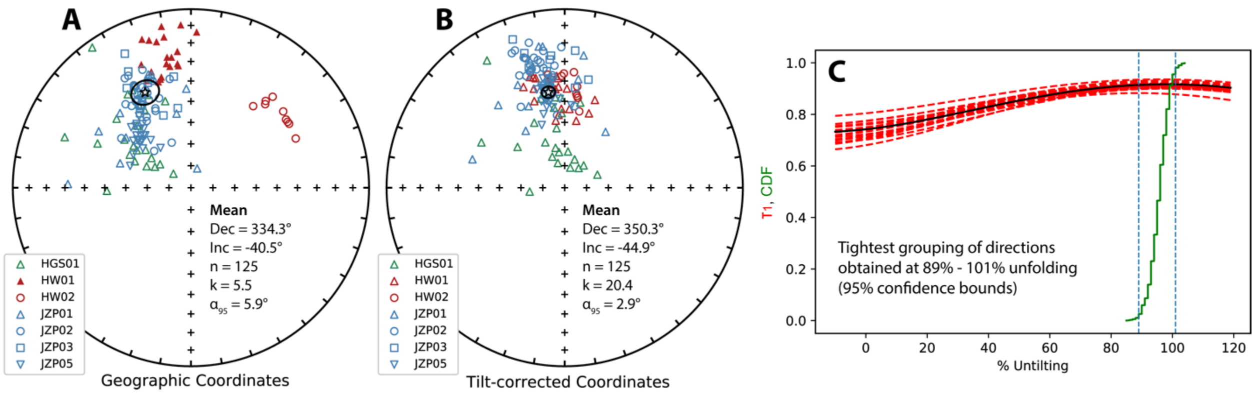

Fig. S8. Specimen directions of HT1 of the seven sills. (**A**) and (**B**)—equal-area plots of HT1 of all 125 specimens from the seven sills. Samples from different sections/sills are shown with different colors/symbols. Filled/open symbols indicate downward/upward directions. Stars indicate the means. (**C**) Results for the Tauxe and Watson (*72*) fold test on HT1 using all specimen directions. Parameters of the fold test are the same as in fig. S6.

Positive baked-contact test on HT1

In the JZP section (fig. S1, fig. S9), sites TR53–54–73, baked siltstones collected from 1–15 cm away from the contact of JZP01, yield a stable high-temperature component (480–550°C) similar to HT1 of JZP01 (fig. S9, fig. S13), interpreted to be a partial thermal remanent magnetization (pTRM) imprinted by the sill. Part of the baked samples shows non-origin-trending decay during ~480–550°C (without change of direction), implying the possibility of a weak residual magnetization associated with the siltstones > 550°C. Nevertheless, fitting great circles to these specimens (between ~480–550°C) does not find another consistent direction near the unblocking temperature (~580°C) but the planes all commonly intersect at HT1 (fig. S10), indicating such a residue, if present in part of the specimens >550°C, is scattered and unstable. Therefore, the line fits between ~480–550°C do not include the origin for pure isolation of the thermal imprint by the sill. We performed a Watson's *V* statistic test (*74*), and the null hypothesis of a common mean for the high-temperature components from JZP01 and the baked siltstones cannot be rejected at 95% confidence level (Watson $V = 2.5$, smaller than the critical value of 6.9).

Samples from sites TR55–56, unbaked siltstones about 30–200 cm below the intrusive contact of the JZP01, do not preserve a stable and consistent high-temperature component. Sites TR87–88, unbaked grey-green siltstones between JZP01–JZP04, yield a stable HT2 that is significantly different from HT1 (fig. S9, fig. S13I). HT2, isolated from ~500–580°C and likely held by magnetite, is a unique NE-up direction that does not resemble any other component found from the Fanjingshan region. It is unclear whether HT2 is a primary detrital remanent magnetization or not without a paleomagnetic confidence test. Collectively, the above results demonstrate a positive baked contact test for HT1, indicating it is a primary thermal remanent magnetization acquired during the cooling of the sills.

The Fanjingshan pole and paleosecular variation test

The seven site-mean directions of the sills in sedimentary coordinates are first converted to corresponding VGPs; averaging the seven VGPs gives the Fanjingshan pole (34.7°S, 118.2°E, $A_{95} = 8.6$) (Fig. 1A, fig. S11A). To test if our paleomagnetic results have sufficiently recorded paleosecular variations of geomagnetic fields, we analyze the VGP angular standard deviation, S_B, after (*75*) and use the n-1 Jackknife resampling method to obtain the confidence intervals of the mean (*76*). After eliminating random errors introduced by the sampling and measuring process, S_B, calculated for the total 7 VGPs, has a mean of 10.4 with uncertainty of 6.3–14.5 (fig. S11B). The 95% confidence intervals of the S_B overlap with the expected VGP dispersion of 11.2–13.3 in Model-G for 0.5–1.5 billion years ago (Ga) at paleolatitude of 25.7° of the mean sampling site at 27.92°N, 108.69°E (fig. S11B). (*25*). We also compare the A_{95} of the Fanjingshan pole with the expected ranges proposed by (*26*) concerning uncertainty arising from secular variation at both the site and specimen level. $A_{95} = 8.6°$ calculated by the seven VGPs of the site-mean directions falls in the critical intervals of 5.5°–24.1° (fig. S11C). $A_{95} = 3.1°$ calculated by all 125 specimens is also within the expected range of 1.7°–3.9° (fig. S11C). These results support that the Fanjingshan pole has sufficient averaging of paleosecular variation of the paleomagnetic fields at ca. 832 Ma.

The validity of the tilt-corrected directions of HT1

Several other lines of evidence corroborate the tilt-corrected directions of HT1. (i) Mafic igneous rocks in the Fanjingshan Gp, including sills and flows, only occur in the lower Fanjingshan Gp and are absent in the whole upper Fanjingshan Gp (*21*, *64*, *67*, *68*). This implies the mafic

magmatism in this area might have already ceased by the upper Fanjingshan Gp deposition when the bedding of the lower Fanjingshan Gp remained horizontal. (ii) No unconformity or depositional hiatus is found within the Fanjingshan Gp and the adjacent formations show conformable contact (*21*, *64*, *67*), suggesting no regional tectonic movements that have tilted the strata of the Fanjingshan Gp prior to regional deformation associated with the Jiangnan Orogen. Together with the positive fold test on HT1, this precludes a possibility that the sills emplaced during the country rocks were tilted rather than horizontal. (iii) The U-Pb LA-ICP-MS 822 ± 8 Ma and ca. 835 Ma youngest populations of detrital zircon (*64, 77*) and the 832.0 ± 8.5 Ma tuff from the upper Fanjingshan Gp (*69*) indicate the depositional age of the upper Fanjingshan Gp may be as young as <832 Ma and imply the emplacement of the ca. 832 Ma sills were likely contemporaneous with part of the Fanjingshan Gp deposition. The evidence above supports the inferred temporal sequence that the mafic sills emplaced before the regional deformation of the Fanjingshan Gp, validating the tilt-correction method and the directions of HT1.

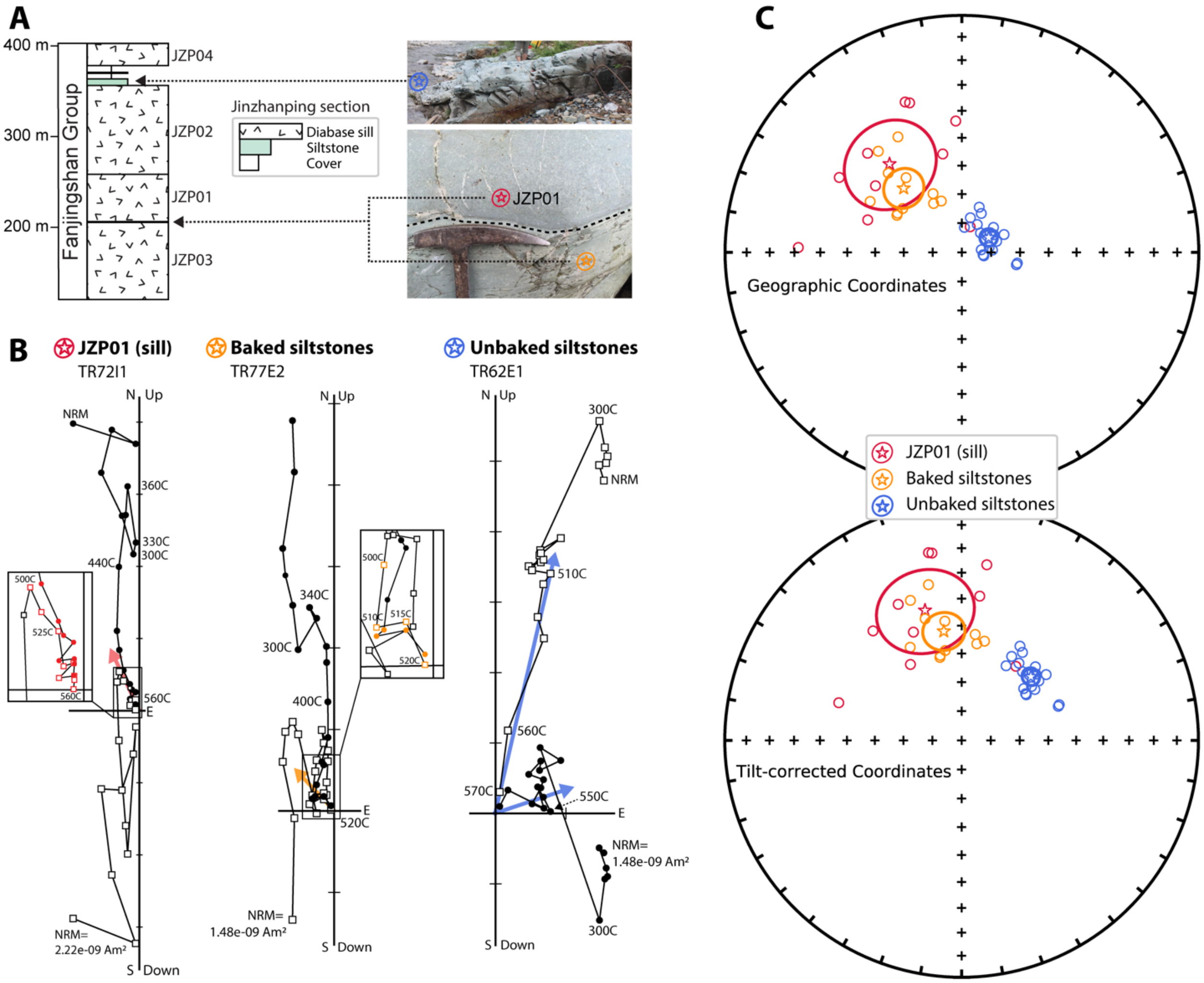

Fig. S9. Baked-contact test for HT1. (**A**) Stratigraphic column indicating sampling localities for the baked-contact test in the Jinzhanping (JZP) section. Legend is the same as in (B). (**B**) Vector component diagrams of demagnetization of representative samples. Each title signifies the rock unit and the specimen. (**C**) Equal-area plots of specimen directions (circles) and site-mean directions with their α_{95} (stars and ellipses). open circles and stars indicate upward directions.

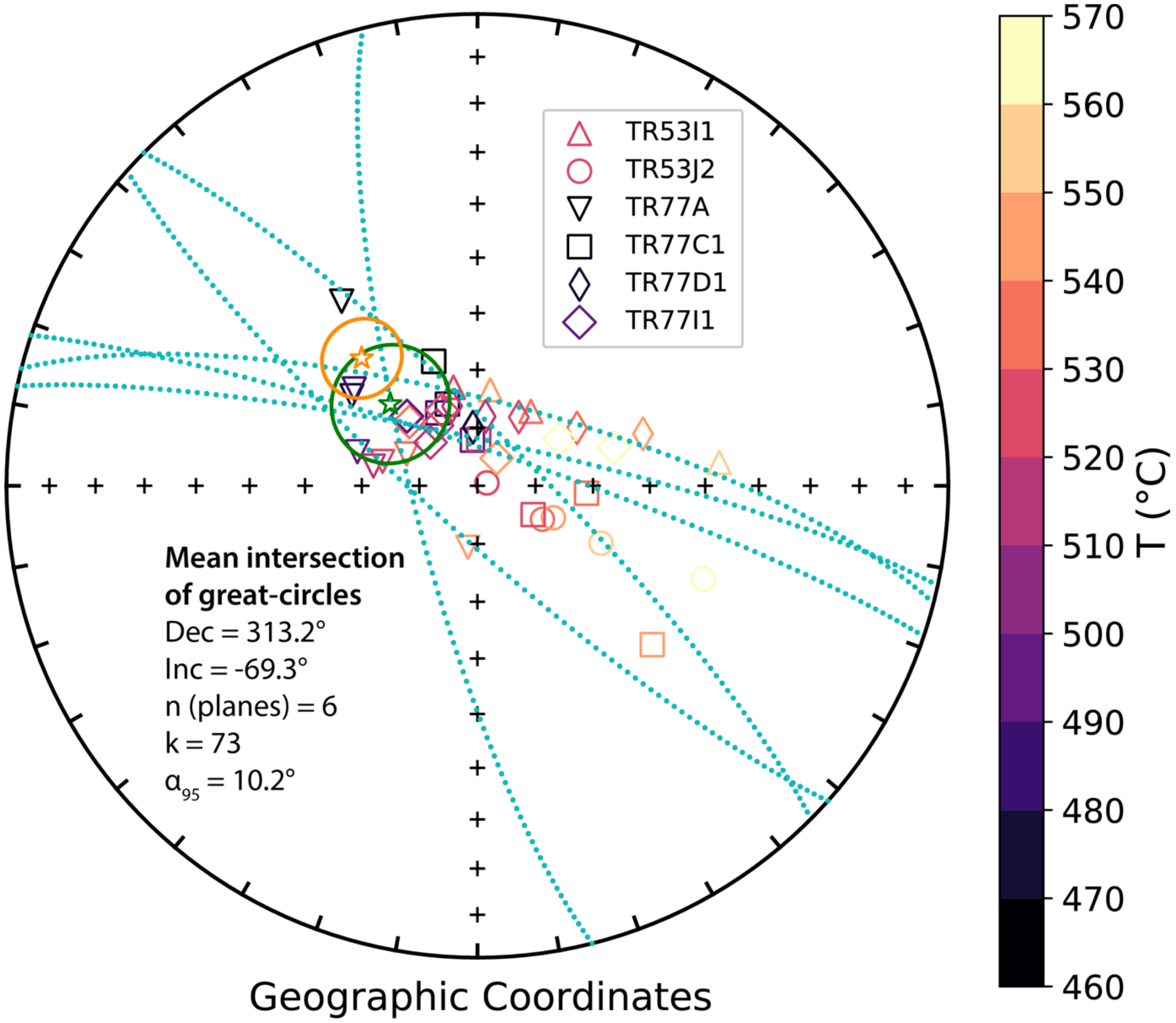

Fig. S10. Fitting great circles to a subset of baked siltstone specimens that show non-origin-trending decay. The green dotted curves show upper-hemisphere planes fitted by the demagnetization data for individual specimens – the color-coded symbols with corresponding demagnetization temperatures (color bar). The green star and ellipse are the mean intersection and α_{95} of the plane fits (n = 6) calculated using the method of McFadden and McElhinny (*78*). The orange star and ellipse exhibit the mean and α_{95} of the line fits for all the baked siltstone specimens (n = 11), as also shown in fig. S9C.

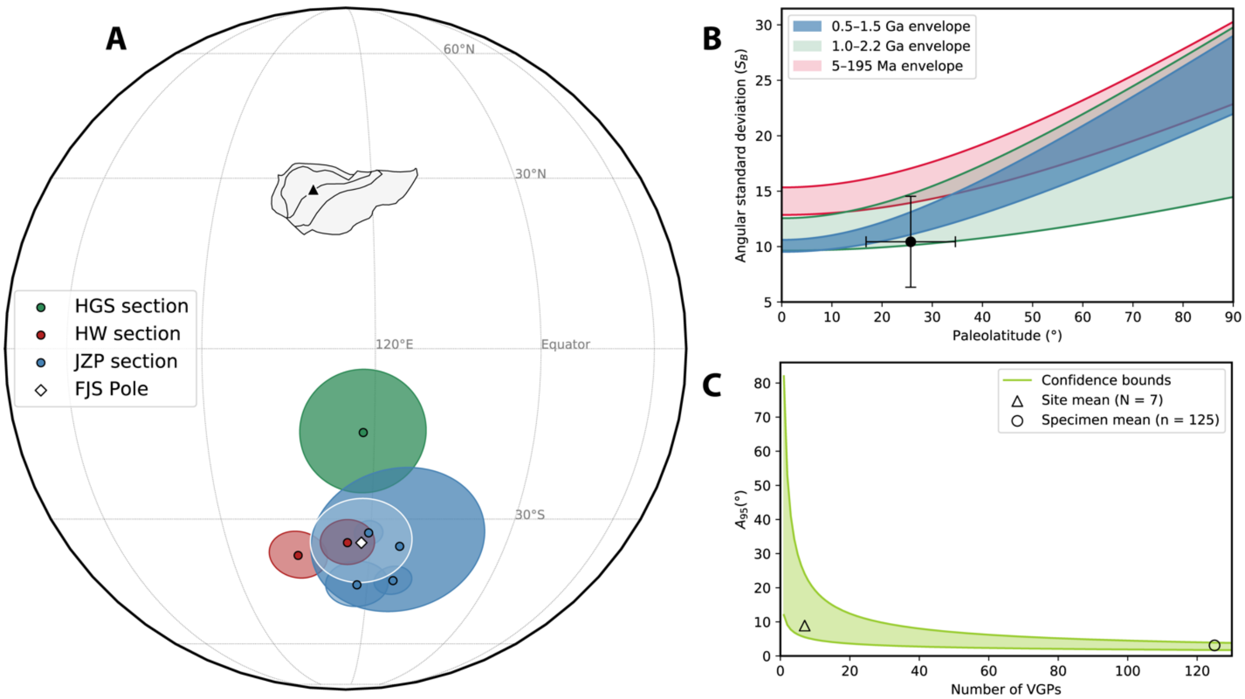

Fig. S11. VGPs of the Fanjingshan pole and paleosecular variation test. (**A**) VGPs of the Fanjingshan (FJS) pole (Table S2). Ellipses show 95% confidence cones of the VGPs/pole (A_{95}). Triangle signifies the mean sampling location of the sills (27.92°N, 108.69°E) (**B**) Angular standard deviation (S_B) (*75*) and its uncertainty compared to paleosecular variation models. Model parameters for 1.0–2.2 Ga, 0.5–1.5 Ga, and 5–195 Ma are from the compilation in (*25*). (**C**) A_{95} calculated by VGPs of site-mean directions (N = 7) and specimen directions (n = 125) compared with the confidence bound suggested by (*26*).

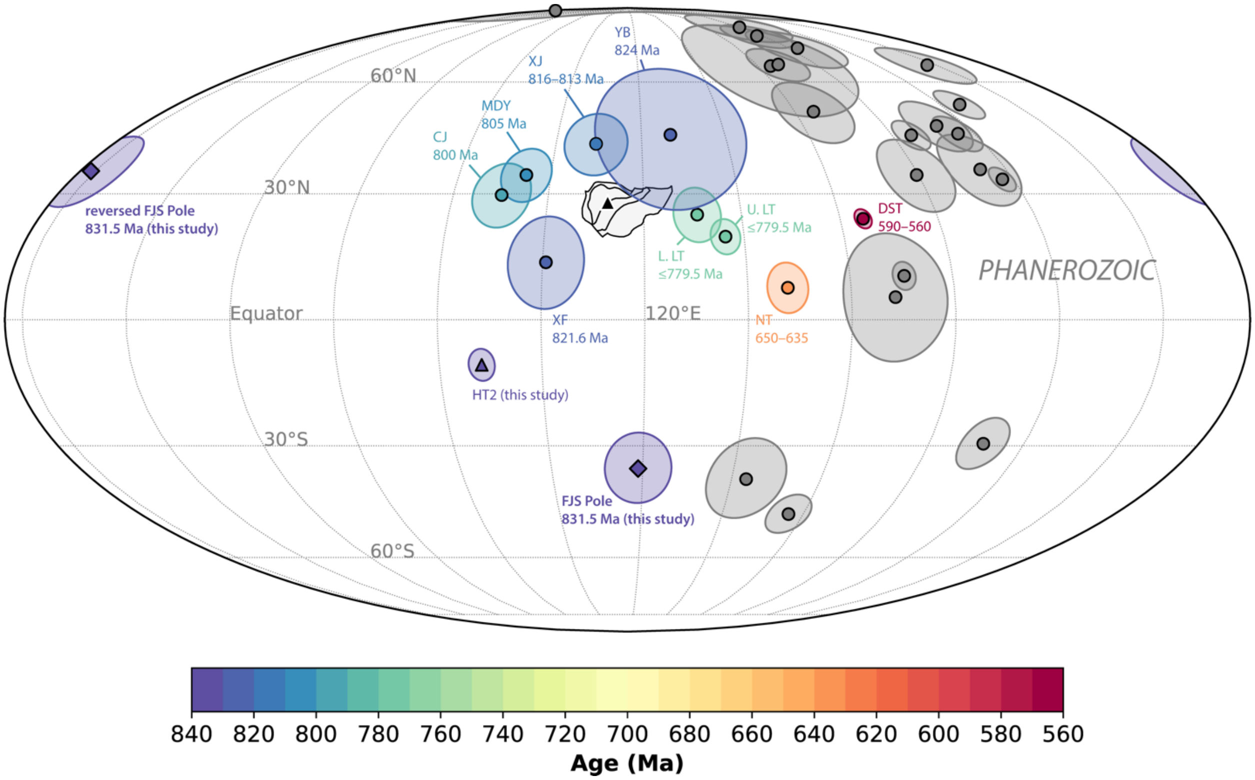

Fig. S12. Apparent polar wander path of South China. Data and references are provided in Table S3 and S5. Coloration according to pole ages is applied to the Neoproterozoic poles; Phanerozoic poles are shown in grey.

Summary of TPW records since the late Mesoproterozoic

Secular TPW is now processing on Earth at ~1.1° Myr^{-1} primarily due to contributions of ongoing glacial isostatic adjustment, quantified by the geodetic observations of relative speed of plate-hotspot motions (*79*). Recent numerical predictions on the present-day TPW rate, which adopt a more accurate ice age rotational theory, suggest a composite nature of the observed rate resulted from both the residual effects of ice age and modern global sea-level rise (*80*).

In the last ~100 Myr, Earth's TPW has been considered to operate in a confined manner of only <6°. The relatively muted TPW magnitude and rate can be ascribed to high mantle viscosity (*81*), a stable and flattened Earth figure introduced by a steady pattern of plate subduction and large-scale mantle upwelling, or remanent bulge stabilization/Earth's excess ellipticity (*1*, *81*, *82*). Recently, paleomagnetic data from Scaglia Rossa limestone of Italy provides evidence for a ~12° TPW oscillation from 86 to 78 Ma, with a mean rate of ~3.0° Myr^{-1} (*9*). The rapid TPW rate was attributed to a larger convective forcing than the normal (*9*). This interpretation is consistent with our predictions for invigorated mantle convective driving that accelerated TPW following the mantle thermal remixing with Pangaea breakup after ca. 170 Ma. The amplitude of TPW was estimated to be 12 ± 3° for single trip, corresponding to 24 ± 6° for the cumulative TPW roundtrip. Thereby, we estimate a mean rate of 3 ± 0.75° Myr^{-1} for the 86–78 Ma TPW oscillations.

Through the rest of the Mesozoic (ca. 250–100 Ma), the TPW rate features a combination of rapid (>1.5° Myr^{-1}) and slow (<1° Myr^{-1}) phases (*7–9*, *54*, *83–85*). A compilation of TPW signals since 320 Ma has been extracted by Torsvik et al. (*7*) from global paleomagnetic data. Four episodes of coherent rotation of all continents around an equatorial axis about the center mass of Pangaea were interpreted: (i) 22.5° CCW (counter-clockwise) between 250 and 200 Ma; (ii) 22.5° CW (clockwise) between 200 and 150 Ma; (iii) 8° CW between 150 and 140 Ma; (iv) 8° CCW between 110 and 100 Ma. Recent analyses on reliable paleomagnetic data from both continental and oceanic plates suggest consistent and higher TPW rates (~1.5–2.1° Myr^{-1}) between ca. 170 and 145 Ma compared to previous estimates (*8*, *54*, *83–85*). The rapid TPW rates of this time interval have been attributed to the cessation of subduction along the western North American margin, long-term accumulation of Mongol-Okhotsk Ocean subducted slabs at high paleolatitudes, stronger global convective forcing, or some combination thereof (*54*, *85*).

Constraints on TPW is more ambiguous in the late Paleozoic. From 400 Ma to 250 Ma, ~60° coherent northward migration of Pangaea at a steady rate of 0.4° Myr^{-1} from the southern latitudes has been recently suggested as evidence for TPW rather than lithosphere-mantle motion (*39*). Considering a ~10° uncertainty in the paleolatitude for Pangaea at 400 Ma and 250 Ma [Fig. 2 from (*39*)], we estimate the rate uncertainty of ± 0.1° Myr^{-1}. The authors also found that the mean TPW rate could be larger (0.5–0.6° Myr^{-1}) using their updated Pangaea reconstruction (*39*). Combining the two sources of uncertainties, we estimate the credible intervals of the 400–250 Ma TPW rate to be 0.3–0.7° Myr^{-1}, with the mean rate at 0.5° Myr^{-1}.

Earlier, a series of late Ordovician to late Devonian TPW events have been inferred from coeval paleomagnetic data from continents including Laurentia, Baltica, Siberia, and Gondwana, displaying two large and opposed shifts of major global plates: a ~50° CCW rotation ca. 450–408 Ma (1.2° Myr^{-1}) followed by a ~25° CW rotation ca. 408–372 Ma (0.7° Myr^{-1}) (*3*, *86*).

However, uncertainties remain in the inferred magnitude and rate of TPW due to the inability to distinguish tectonic-motion components from the total apparent polar wander path of Gondwana, during the closure of the Rheic Ocean and amalgamation between Gondwana and Laurussia (*7*). Accordingly, Torsvik et al. (*7*) considered a maximum TPW rate at ~2.0° Myr^{-1} at 430–410 Ma interpreted for a synthesized apparent polar wander path for Laurentia, concerning a portion of the pole shift was allocated to plate-tectonic movement. Taking their reported great-circle difference (GCD) as an approximation to the pole uncertainty and using the standard uncertainty propagation, we quantify the means and uncertainties of the maximum TPW rates using their original data [Table. 5 from Torsvik et al. (*7*)] that yield 1.9 ± 0.7° Myr^{-1} between 430 and 420 Ma and 2.0 ± 0.6° Myr^{-1} between 420 and 410 Ma.

Late Ediacaran to Cambrian TPW records (555–505 Ma) have been proposed from Laurentia and Gondwana (*87*), among which the most recent robust data set came from Australia (*88*). According to updated paleomagnetic results from the Amadeus Basin, central Australia, the paleogeographic shift of assembled Gondwana was ~60° over ~40 Myr, with plate velocity estimates from 8–28 cm yr^{-1}, inferred to be either nonuniformitarian plate tectonics or an episode of rapid TPW, or both (*88*). The Early Cambrian TPW hypothesis has been interpreted as consistent with coeval global $\delta^{13}C_{carb}$ variations and exceptionally fast evolutionary rates in the biosphere (a factor of 20 higher), both of which were potentially triggered by prominent paleogeographic shifts of global continents and sea-level fluctuations due to large-amplitude and long-term TPW (*87*, *89*).

Earlier in the Ediacaran, ca. 615–565 Ma rapid and, potentially, oscillatory TPW has been postulated based on paleomagnetic data from both sedimentary and igneous rocks in Laurentia, West Gondwana, Baltica, and Australia (*14*). A ~90° CW rotation of these continents between ca. 615 and 590 Ma is followed by an opposite ~90° rotation around a similar TPW axis from 575 to 565 Ma (*14*). From global data, TPW mean rate estimates for the two TPW phases were recently proposed at ~3 ± 1° Myr^{-1} and ~10° Myr^{-1}, respectively (*14*). Notably, the ~10° Myr^{-1} estimate for the latter 575–565 Ma event comes from averaging very scattered results from various continents, which also have very large uncertainties (error bars from +/- 2° Myr^{-1} to +/- 15° Myr^{-1}) (*14*). Robert et al. (*15*) evaluated Ediacaran TPW mechanisms with global geographic reconstructions and a mantle dynamics model. They found that the reactivation of a girdle of subduction surrounding the continents followed by a reduced return flow could potentially explain the observed TPW oscillation. The modeled mean TPW velocities are consistent between the two TPW shifts: ~2.9° Myr^{-1} and 3.2–3.5° Myr^{-1} for 615–590 Ma and 575–565 Ma, respectively. For the 575–565 Ma event, although the mean TPW rate estimate from observations (~10° Myr^{-1}) is larger than the modeled mean values (3.2–3.5° Myr^{-1}), they nevertheless overlap each other within uncertainty. Therefore, we regard the modeled speed of 3.2–3.5° Myr^{-1} from (*15*) as a reasonable and likely estimate, while ~10° Myr^{-1} is a possibility of a maximum speed of this interval. Alternatively, these large Ediacaran pole shifts have been interpreted to record non-Geocentric Axial Dipole (GAD) fields with extremely weak geomagnetic moments and rapid reversals (*90*, *91*).

In the Tonian, during the Bitter Springs isotopic stage (810–795 Ma), three consecutive paleomagnetic poles from the carbonate units of the Akademikerbreen Group, East Svalbard, Norway, exhibit two nearly opposite shifts in the direction of ~83° total rotation within ~15 Myr

(*10*). These shifts in pole position broadly coincide with changes in $\delta^{13}C_{carb}$ and local sea level, supporting a TPW interpretation (*10*). The age of the three Svalbard paleomagnetic poles that bracket the Bitter Springs isotopic stage were constrained by chemostratigraphic correlations with the sedimentary sessions in Canada and Ethiopia, in which absolute dating were obtained (*11*, *92*, *93*). Using these age constraints, the Svalbard poles yield a TPW rate of ≥40–54 cm yr^{-1} (≥3.6–4.9° Myr^{-1}) for the whole Bitter Springs Stage (from ca. 810 to <795 Ma) (*11*). Using a Monte Carlo approach accounting for the uncertainties between pole ages, Park et al. (*12*) determine the TPW rate to be ≥60 cm yr^{-1} (≥5.4° Myr^{-1}) between the pre-Bitter Springs pole (> ca. 810 Ma) and syn-Bitter Springs pole (ca. 810–795 Ma). From South China, the newly reported Xiajiang pole (816–810 Ma, pre-Bitter Springs isotopic stage) (*12*) and Madiyi pole (ca. 805 Ma, syn-Bitter Springs isotopic stage) (*32*) constrain pole motion significantly less than that predicted from the coeval Svalbard poles, pointing to the possibility of TPW rotation counteracted by tectonic motion of South China.

Preceding the Bitter Springs Stage, the ca. 832 Ma Fanjingshan pole from this study and the ca. 821 Ma Xiaofeng pole (*28*, *29*) constrain the total pole motion of South China to be 54.7 ± 13.9° at an estimated rate of 3.5–7.8° Myr^{-1} resulting from pure TPW (accounting for possible tectonic movements). If using the proposed revised Xiaofeng pole from Jing et al. (*94*), the total pole motion between 832 and 821 Ma would be 69.8 ± 16.9° at a rate of 7.1 ± 1.7° Myr^{-1}, agreeing with the results using the original Xiaofeng pole (*28*) within uncertainty. This large low-to-high-latitude motion of South China at 832–821 Ma is comparable to the ~56° pole-to-equator latitude change of Baltica in an overlapping period (Fig. 1C–D), determined by the 848 ± 27 Ma (^{40}Ar–^{39}Ar biotite plateau date) (*35*)/834 ± 9 Ma (Rb–Sr isochron age) (*95*) Hunnedalen dykes pole (*35*) and ca. 836–803 Ma Katav Formation pole and Inzer Formation pole (*16*, *17*) loosely constrained by Rb-Sr illite isochron dates from shale in the Inzer Formation (*96*). As Baltica has been established as part of the assembled Rodinia in the Tonian (*10*, *36*), the coherent motion of Baltica (assembled Rodinia) and possibly disconnected South China reinforce the hypothesis that these motions recorded TPW.

Before the Neoproterozoic, it has been suggested that TPW may account for, at least in part, the rapid plate velocity (~27–34 cm yr^{-1}) implied by the Keweenawan Track between ca. 1,110 and 1,080 Ma obtained from the Midcontinent Rift, Laurentia (*56*). With the implementation of the Bayesian approach, Swanson-Hysell et al. (*56*) have evaluated different models to disentangle plate-tectonic and TPW components of the 1,110–1,080 Ma apparent polar wander path. The TPW rate could be feasibly interpreted to be 5–22 cm yr^{-1} accompanied by a fast plate-tectonic motion of Laurentia at ~15 cm yr^{-1}. Alternatively, the Keweenawan Track can potentially be explained entirely as plate tectonic motion, with TPW close to zero (*56*). Similar rapid pole motions of this stage are supported by possibly contemporaneous paleomagnetic results from carbonate successions of the Nanfen Formation, North China, that show significant pole-to-equator latitudinal change consistent with the data from Laurentia (*97*). Nevertheless, the timing of these pole shifts is poorly constrained without direct and precise age controls on these carbonate units (*97*).

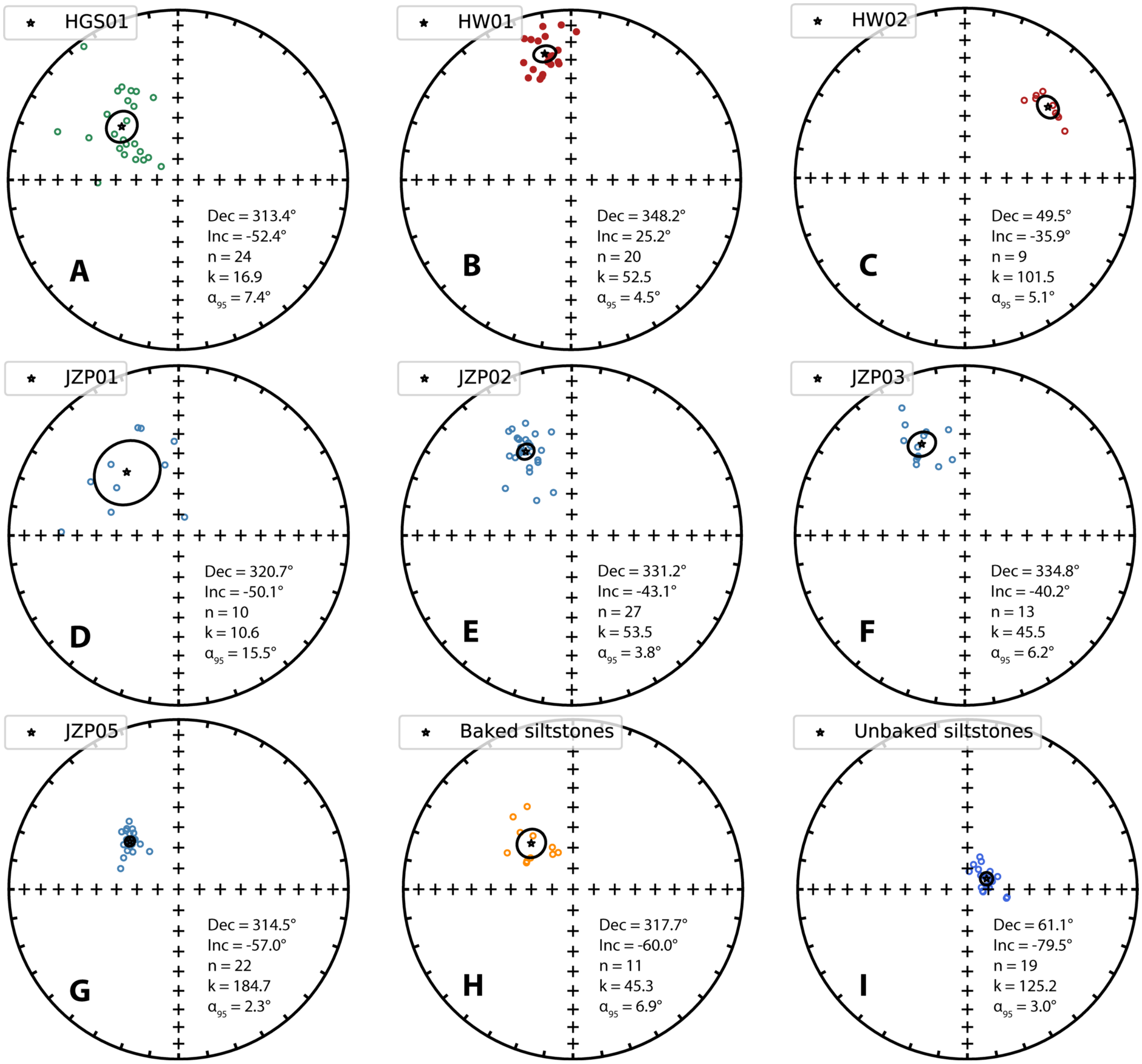

Fig. S13. HT1 of individual sills and sites of country rocks in geographic coordinates. Filled symbols indicate downward directions; open symbols indicate upward directions. Circles indicate specimen directions; Stars and associated ellipse are the site-mean directions and 95% confidence cones (α_{95}).

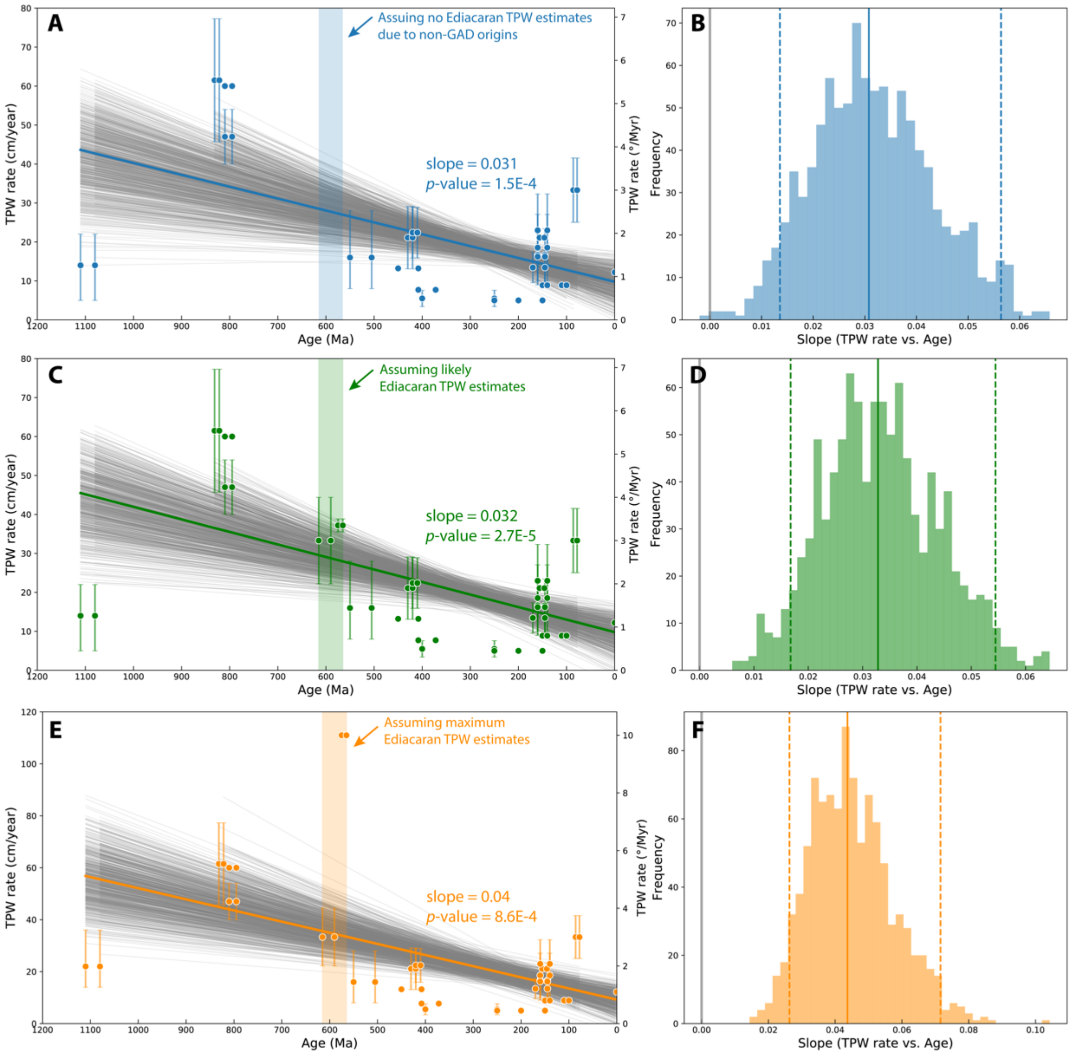

Fig. S14. Bootstrap linear regression models of the TPW rate as a function of time with sensitivity tests. (**A**)&(**C**)&(**E**) Linear regression using case-resampling bootstrap. Dots with error bars are original data points compiled in Table S8. For the analysis, observations are randomly selected from the original data set (with replacement) to form 1,000 resampled data sets of the same sample size. For each re-sampled data set, the best-fit slope is determined and plotted (grey line). The three cases (A), (C), and (E) demonstrate a set of sensitivity tests: (A) assuming non-GAD origins of the rapid pole shifts during the Ediacaran and therefore no estimates of TPW of this time; (B) assuming the likely and conservative Ediacaran TPW rate estimates consistent with those modeled by Robert et al. (*15*); (C) assuming the possibility of maximum TPW speeds (~10° Myr^{-1} between 575 and 565 Ma) implied from Robert et al. (*14*). TPW rate (° Myr^{-1}) is translated to the corresponding plate velocity (cm yr^{-1}) at a point 90° from the TPW axis using a factor of 11.1. (**B**)&(**D**)&(**F**) Bootstrap distribution of the slope estimates using case-resampling corresponding to (A)&(C)&(E). For all the three cases, the 95% confidence intervals of the slope estimates overlap each other and do not envelop slope = 0. These results suggest that the positive correlation of TPW rate vs. age (long-term decrease of TPW rate with time) is (i) significant at 95% confidence level and (ii) independent from the variably interpreted rapid TPW rates or their presence in the Ediacaran.

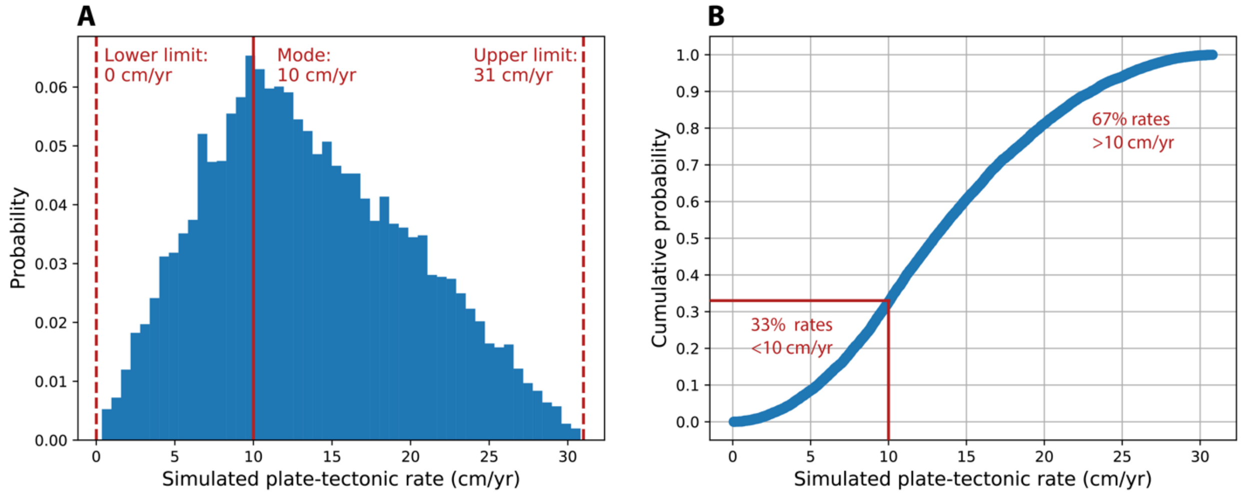

Fig. S15. Distribution of the simulated plate-tectonic rates of South China between 832 and 821 Ma. (**A**) Probability density function (PDF) of the simulated rates using a triangular distribution assuming (1) the mode at 10 cm yr^{-1} that corresponds to the fastest plate velocity of South China extracted from the Neoproterozoic plate kinematic models (*55*); (2) the lower limit at 0 cm yr^{-1}; (3) the upper limit at ~31 cm yr^{-1} that represents the fastest tectonic movement implied since the late Mesoproterozoic suggested from the rapid plate motion of Laurentia between 1,110 and 1,080 Ma (*56*). (**B**) Cumulative density function (CDF) of the simulated rates. It shows that most cases (~67%) place the simulated rates above 10 cm yr^{-1}, weighted toward the endmember of very high tectonic velocities. Therefore, the distribution adequately accommodates large effects of potential tectonic motions.

Table S1. Summary of CA-ID-TIMS U-Pb data for TS01C

	Dates (Ma)							Composition					Isotopic Ratios								
Fraction	$^{206}Pb/^{238}U$ (a)	±2σ	$^{207}Pb/^{235}U$ (b)	±2σ	$^{207}Pb/^{206}Pb$ (a)	±2σ	% disc (c)	U (pg)	Th/U (d)	Pb* (pg) (e)	Pb_c (pg) (f)	Pb^*/Pb_c (g)	$^{206}Pb/^{204}Pb$ (h)	$^{208}Pb/^{206}Pb$ (i)	$^{206}Pb/^{238}U$ (i), (a)	±2σ %	$^{207}Pb/^{235}U$ (i)	±2σ %	$^{207}Pb/^{206}Pb$ (i), (a)	±2σ %	Corr. coef.
z1	831.29	1.29	833.10	1.39	837.94	3.27	0.82	794.01	1.19	134.70	1.14	118.47	6037.73	0.36386	0.13764	0.16569	1.27159	0.2439	0.06704	0.15359	0.77067
z2	831.71	0.56	832.28	0.86	833.83	1.86	0.28	1077.61	1.20	183.41	0.29	624.49	31661.8	0.36793	0.13771	0.07195	1.26976	0.15168	0.0669	0.08309	0.92646
z3	831.22	0.55	831.74	0.81	833.12	1.77	0.25	1044.81	1.04	171.28	0.32	539.31	28372.1	0.31637	0.13763	0.07021	1.26855	0.14234	0.06688	0.07857	0.89834
z4	832.56	0.54	832.31	0.86	831.62	2.06	-0.09	573.19	1.27	99.13	0.36	276.76	13834.8	0.38936	0.13786	0.06967	1.26981	0.15201	0.06683	0.09321	0.86001
z5	831.66	0.59	831.89	0.86	832.50	1.81	0.12	1236.80	1.24	211.98	0.30	697.03	35090.5	0.37801	0.1377	0.07532	1.26888	0.15072	0.06686	0.08039	0.91909

$^{206}Pb/^{238}U$	Uncertainty (2σ)					
date (Ma)	*X*	*Y*	*Z*	MSWD	*n*	*N*
831.51	0.32	0.46	1.00	0.65	4	5

Notes :

Colored fractions indicate zircons used in age calculation.

X—internal (analytical) uncertainty in the absence of all external or systematic errors; Y—incorporates the U-Pb tracer calibration error; Z—includes X and Y, as well as the uranium decay constant errors.

MSWD—mean square of weighted deviates; *n* —number of analyses included in age calculation; *N* —total number of zircon analyses.

(a) Corrected for initial Th/U disequilibrium using radiogenic 208Pb and Th/U[magma] = 2.8.

(b) Isotopic dates calculated using λ238 = 1.55125E-10 (*100*) and λ235 = 9.8485E-10 (*98*).

(c) % discordance = 100 - (100 * (206Pb/238U date) / (207Pb/206Pb date))

(d) Th contents calculated from radiogenic 208Pb and 230Th-corrected 206Pb/238U date of the sample, assuming concordance between U-Pb and Th-Pb systems.

(e) Total mass of radiogenic Pb.

(f) Total mass of common Pb.

(g) Ratio of radiogenic Pb (including 208Pb) to common Pb.

(h) Measured ratio corrected for fractionation and spike contribution only.

(i) Measured ratios corrected for fractionation, tracer and blank.

Table S2. Site-mean directions of the mafic sills and country rocks in the Fanjingshan Group

Site ID	Location	*n* / *N*	Bedding		In situ		Tilt-corrected								
			Dip dir (°)	Dip (°)	*Dg* (°)	*Ig* (°)	*Ds* (°)	*Is* (°)	*k*	α_{95} (°)	λ (°S)	ϕ (°E)	*dp* / *dm* (°)	A_{95} (°)	Plat (°)
**Huguosi section (HGS)**															
HGS01	27°54'56''N, 108°38'8''E	24	271.2	23.0	313.4	-52.4	346.6	-64.9	16.9	7.4	14.2	118.0	9.6/11.9		46.9
**Heiwan section (HW)**															
HW01	27°52'12''N, 108°44'47''E	20	330.0	74.6	348.2	25.2	353.9	-45.7	52.5	4.5	34.7	115.4	3.7/5.7		27.1
HW02	27°51'52''N, 108°45'36''E	9	110.0	51.5	49.5	-35.9	3.4	-42.6	101.5	5.1	37.3	104.9	3.9/6.3		24.7
**Jingzhanping section (JZP)**															
JZP01	27°52'17''N, 108°37'16''E	10	223.9	23.3	320.7	-50.1	344.3	-42.4	10.6	15.5	35.5	126.2	11.8/19		24.5
JZP02	27°52'14''N, 108°37'15''E	27	220.7	22.6	331.2	-43.1	346.8	-32.2	53.3	3.8	42.9	125.9	2.4/4.3		17.5
JZP03	27°52'25''N, 108°37'22''E	13	237.4	25.4	334.8	-40.2	353.2	-32.7	45.5	6.2	43.9	117.6	4.0/7.0		17.8
JZP05	27°51'57''N, 108°37'2''E	22	223.0	27.7	314.5	-57.0	349.5	-47.4	184.7	2.3	32.7	119.6	1.9/3.0		28.5
**Mean of site-means**		**7**					**351.3**	**-44.1**	**46.5**	**8.9**					**26.7**
**The Fanjingshan pole**		**7**							**50.7**		**34.7**	**118.2**		**8.6**	
Baked siltstones	27°52'17''N, 108°37'16''E	11	223.9	23.3	317.7	-60.0	350.7	-51.5	45.3	6.9	29.3	117.6	6.4/9.4		32.2
Unbaked siltstones	27°52'14''N, 108°37'15''E	19	220.7	22.6	61.1	-79.5	47.5	-57.4	125.2	3.0	10.6	72.4	3.2/4.4		38.0

Notes : *n* / *N* : number of sample for statistics/number of sites (rock units) for statistics ; *Dg* -*Ig* / *D* s-*Is* —declination and inclination in geographic/tilt-corrected coordinates; *k*—precision parameter of Fisher (*61*); α_{95}—radius of 95% confidence circle of the fisher mean; λ and ϕ—latitude and longitude of virtual geomagnetic pole (VGP) in present-day geographic coordinates; dp/dm—semiminor and semimajor axes of the 95% polar error ellipse; A_{95}—radius of 95% confidence circle of paleomagnetic pole; Dip dir—dip direction; Plat—paleolatitude.

Table S3. Neoproterozoic paleomagnetic poles for South China

Pole ID	Age (Ma)	Rock unit	λ (°N)	ϕ (°E)	(*dm* /*dp*)/A95 (°)	References
DST	~590 to 560	Doushantuo Formation (Member 3)	23.9	187	3.0/1.8	(*99*)
NT	~650 to 635	Nantuo Formation	7.5	161.6	5.9	(*100*)
U. $LT_{0.6}$	≤779.52 ± 0.92	Upper Liantuo Formation (Member 2)	19.6	144.4	4.2	(*101* , *12*)
L. $LT_{0.6}$	≤779.52 ± 0.92	Lower Liantuo Formation (Member 1)	24.9	136.4	6.6	(*102* , *12*)
$CJ_{0.6}$	799.5 ± 8.4	Chengjiang Formation	29.7	75.3	7.9	(*94* , *12*)
$MDY_{0.6}$	804.90 ± 0.99	Madiyi Formation	34.7	82	6.7	(*32* , *12*)
$XJ_{0.6}$	816–810	Xiajiang Formation	42.7	104	8.1	(*12*)
XF	821.64 ± 0.2	Xiaofeng dykes	13.5	91	11.3/10.5	(*28* , *29*)
YB	824 ± 6	Yanbian dykes	45.1	130.4	19	(*103*)
**FJS**	**831.51 ± 0.32**	**Fanjingshan sills**	**-34.7**	**118.2**	**8.6**	**this study**

Notes: The *0.6* notation—paleomagnetic poles after f = 0.6 inclination correction; λ and ϕ—latitude and longitude of paleomagnetic poles in present-day geographic coordinates; *dp/dm* —semiminor and semimajor axes of 95% polar error ellipse; A_{95}—radius of 95% confidence cone of paleomagnetic pole.

Table S4. 850–810 Ma paleomagnetic poles for Rodinia

Pole ID	Age (Ma)	Rock unit	**λ** (°N)	**ϕ** (°E)	(*dm* /*dp*)/A95 (°)	Pole references	Age references
East Svalbard							
L. Grus	~820–810	Lower Grusdievbreen Formation	19.6	204.9	10.9	(*10*)	(*10–12*)
Baltica							
K. Fm	~836–803	Katav Formation	35.9	168.4	1.5/1.9	*(17)*	*(96)*
I. Fm	~836–803	Inzer Formation	27.7	185.9	4.6/9.2	*(17)*	*(96)*
HD	848 ± 27/834 ± 9	Hunnedalen dykes	-41	222	10.5	*(35)*	*(35, 95)*
West Africa							
MD	[illegible]57.2 ± 8.5 & 855 ± 16	Manso dykes	-28.3	177.6	12.7	*(37)*	*(37)*

Notes: λ and ϕ—latitude and longitude of paleomagnetic poles in present-day geographic coordinates;
dp/dm —semiminor and semimajor axes of 95% polar error ellipse; A_{95}—radius of 95% confidence cone of the paleomagnetic pole.

Table S5. Phanerozoic paleomagnetic poles for South China

Pole ID	Age	**λ (°N)**	**ϕ (°E)**	**(*dm* /*dp*)/A_{95} (°)**	References
Cz	Cenozoic	87.5	315.6	5.9	(*104*)
K_2	Late Cretaceous	75.5	205.3	4.5	(*104*)
K_1	Early Cretaceous	79	208.3	5.9	(*104*)
J_3	Late Jurassic	71	215.1	6.7	(*104*)
J_2	Middle Jurassic	65	186.2	15	(*105*)
J_1	Early Jurassic	65.5	190.9	5.3	(*106*)
T_3	Late Triassic	51.4	187.2	8.5	(*104*)
T_2	Middle Triassic	34.7	209.4	9.1	(*104*)
T_1	Early Triassic	45	216.8	3.9	(*104*)
P_2	Permian - Guadalupian & Lopingian	53.4	247.7	4	(*104*)
P_1	Permian - Cisuralian	65.3	265.2	8.1/4.2	(*107*)
C_1	Carboniferous - Mississippian	47.5	229.1	9.6/4.8	(*108*)
D_3	Late Devonian	45.4	234.1	6.6/3.3	(*108*)
D_2	Middle Devonian - Givetian	33.6	236.4	3	(*109*)
D_{12}	Early & Middle Devonian	36.1	231.4	12.5/6.5	(*108*)
S_{23}	Siurian - Wenlock & Ludlow	10.3	195.8	3.3	(*110* , *111*)
O_{23}	Middle & Late Ordovician	−29.5	227.1	6.3	(*113*)
O_1	Early Ordovician	−38.4	154.9	14.2/7.4	(*107*)
Cam_2	Cambrian - Series 2	−47.6	174.5	5.2	(*112* , *113*)
Cam_1	Cambrian - Series 2	5.3	192.7	15	(*113*)

Notes: λ and ϕ—latitude and longitude of paleomagnetic pole in present-day geographic coordinates; dp/dm—semiminor and semimajor axes of 95% polar error ellipse; A_{95}—radius of 95% confidence circle of the paleomagnetic pole.

Table S6. Euler rotations for paleogeographic model of South China between 832 and 780 Ma (Fig. 1B)

Pole name	Age	Rotation relative to	λ (°N)	ϕ (°E)	Rotation (°)	References
U. $LT_{0.6}$	≤779.52 ± 0.92	Spin axis	-25.2	32.2	76.5	(*12*)
L. $LT_{0.6}$	≤779.52 ± 0.92	Spin axis	-23.6	24.4	69.1	(*12*)
$CJ_{0.6}$	799.5 ± 8.4	Spin axis	-9.0	-13.8	54.7	(*12*)
$MDY_{0.6}$	804.90 ± 0.99	Spin axis	-0.9	-2.7	50.9	(*12*)
$XJ_{0.6}$	816–810	Spin axis	1.4	6.8	52.6	(*12*)
XF	821.64 ± 0.2	Spin axis	-21.0	-16.7	83.1	this study
FJS	831.51 ± 0.32	Spin axis	12.2	-176.6	-131.1	this study

Notes: λ and ϕ—latitude and longitude of the euler pole in present-day geographic coordinates. Euelr rotation of South China 821–780 Ma follows the preferred 'minimize difference' model in (*12*). Euler rotations are relative to spin axis (mantle).

Table S7. Euler rotations for 832–821 Ma Rodinia paleogeographic model (Fig. 1C–D)

Plate name	Plate ID	Rotation relative to	Age	***λ*** **(°N)**	***ϕ*** **(°E)**	**Rotation (°)**	**References**
Laurentia	L	Spin axis	832	-8.7	-81.9	-117.9	this study
Laurentia	L	Spin axis	821	-37.0	-97.0	-91.2	this study
South China	SC	Laurentia	832–821	78.1	77.7	54.1	this study
East Svalbard	ES	Laurentia	832–821	-81.0	125.0	68.0	(*10* , *12*)
Baltica	Ba	Laurentia	832–821	81.5	-110.0	-50.0	(*114*)
India	Ind	South China	832–821	20.9	63.2	-67.9	(*33*)
Lhasa	Lh	South China	832–821	24.9	77.5	-77.4	(*33*)
South Qiangtang	SQ	South China	832–821	16.1	68.8	-68.1	(*33*)
Greenland	G	Laurentia	832–821	68.0	-119.0	-14.0	(*10*)
Siberia-Aldan	Si-Al	Siberia-Anabar	832–822	60	115	25	(*115*)
Siberia-Anabar	Si-An	Laurentia	832–821	77	98	137	(*115*)
North China	NC	Laurentia	832–821	58.0	30.0	68.0	(*116*)
North Australia	NA	Laurentia	832–821	64.0	113.0	108.0	(*116*)
South Australia	SA	North Australia	832–821	-20.0	135.0	40.0	(*117*)
Mawson	Ma	South Australia	832–821	-3.9	37.9	30.9	(*12*)
Kalahari	K	Laurentia	832–821	-7.1	-37.1	-181.7	(*55*)
Rio de la Plata	RDLP	Laurentia	832–821	-0.1	120.0	148.5	(*55*)
Amazonia	Am	Laurentia	832–821	12.0	-46.0	-110.6	(*55*)
Parana	Pp	Amazonia	832–821	0.7	103.2	-30.8	(*55*)
West Africa	WA	Baltica	832–821	1.4	15.2	-66.1	(*37*)

Notes: λ and ϕ—latitude and longitude of the euler pole in present-day geographic coordinates.

Table S8. Probable TPW rate estimates since the late Mesoproterozoic

Age (Ma)	Era	Period	TPW rate (°/Myr)	Uncertainty (+/-)	References
1110–1080	Late Mesoproterozoic	Stenian	1.3	0.7/0.8	(*56*)
832–821	Neoproterozoic	Tonian	5.5[(1)]	1.4	**this study**
832–821	Neoproterozoic	Tonian	5.5[(2)]	2.3/2.0	**this study**
810–795	Neoproterozoic	Tonian	≥4.2	0.6	(*11*)
810–795	Neoproterozoic	Tonian	≥5.4		(*12*)
615–590	Neoproterozoic	Ediacaran	3	1	(*14*)
575–565	Neoproterozoic	Ediacaran	3.35[(3)]	0.15	(*15*)
575–565	Neoproterozoic	Ediacaran	10[(4)]		(*14*)
550–505	Paleozoic	Ediacaran–Cambrian	1.4	1.1/0.7	(88)
450–408	Paleozoic	Odovician–Silurian	1.2		(*3*, *86*)
430–420	Paleozoic	Silurian	1.9	0.7	(*7*)
420–410	Paleozoic	Devonian	2	0.6	(*7*)
408–372	Paleozoic	Devonian	0.7		(*3*, *86*)
400–250	Paleozoic	Devonian–Triassic	0.5	0.2	(*39*)
250–200	Mesozoic	Triassic	0.45		(*7*)
200–150	Mesozoic	Jurassic	0.45		(*7*)
170–145	Mesozoic	Jurassic	1.21	0.35	(*85*)
160–145	Mesozoic	Jurassic	1.46	0.65	(*85*)
160–145	Mesozoic	Jurassic	1.5		(*8*)
156–146	Mesozoic	Jurassic	1.9		(*84*)
160–140	Mesozoic	Jurassic–Cretaceous	1.67	0.77	(*83*)
160–140	Mesozoic	Jurassic–Cretaceous	2.07	0.84	(*83*)
150–140	Mesozoic	Jurassic–Cretaceous	0.8		(*7*)
110–100	Mesozoic	Cretaceous	0.8		(*7*)
86–78	Mesozoic	Cretaceous	3	0.75	(*9*)
0	now		1.1		(79)

Notes : [(1)]Considering no plate tectonic movements. [(2)]Accounting for plate tectonic movements.
[(3)]Conservative estimate of this period. [(4)]Maximum estimate of this period.

Data file S1. Fits of high-temperature components. *Dg*/*Ig*: declination and inclination in geographic coordinates. MAD: maximum angle of deviation. Fitting method signifies fits forced/unforced to pass through the origin. Fitting steps correspond to the demagnetizing steps as recorded in the measurement-level data files.

REFERENCES AND NOTES

1. I. Matsuyama, F. Nimmo, J. X. Mitrovica, Planetary reorientation. *Annu. Rev. Earth Planet. Sci.* **42**, 605–634 (2014).

2. V. Tsai, D. Stevenson, Theoretical constraints on true polar wander. *J. Geophys. Res. Solid Earth* **112**, B05415 (2007).

3. J. R. Creveling, J. X. Mitrovica, N.-H. Chan, K. Latychev, I. Matsuyama, Mechanisms for oscillatory true polar wander. *Nature* **491**, 244–248 (2012).

4. I. Rose, B. Buffett, Scaling rates of true polar wander in convecting planets and moons. *Phys. Earth Planet. Inter.* **273**, 1–10 (2017).

5. T. D. Raub, J. L. Kirschvink, D. A. D. Evans, True polar wander: Linking deep and shallow geodynamics to hydro- and biospheric hypotheses, in *Treatise on Geophysics* (Elsevier, 2007), pp. 565–589.

6. B. Steinberger, T. Torsvik, Absolute plate motions and true polar wander in the absence of hotspot tracks. *Nature* **452**, 620–623 (2008).

7. T. H. Torsvik, R. Van der Voo, U. Preeden, C. Mac Niocaill, B. Steinberger, P. V. Doubrovine, D. J. J. van Hinsbergen, M. Domeier, C. Gaina, E. Tohver, J. G. Meert, P. J. A. McCausland, L. R. M. Cocks, Phanerozoic polar wander, palaeogeography and dynamics. *Earth-Sci. Rev.* **114**, 325–368 (2012).

8. D. V. Kent, B. A. Kjarsgaard, J. S. Gee, G. Muttoni, L. M. Heaman, Tracking the Late Jurassic apparent (or true) polar shift in U-Pb-dated kimberlites from cratonic North America (Superior Province of Canada). *Geochem. Geophys. Geosyst.* **16**, 983–994 (2015).

9. R. N. Mitchell, C. J. Thissen, D. A. Evans, S. P. Slotznick, R. Coccioni, T. Yamazaki, J. L. Kirschvink, A Late Cretaceous true polar wander oscillation. *Nat. Commun.* **12**, 3629 (2021).

10. A. C. Maloof, G. P. Halverson, J. L. Kirschvink, D. P. Schrag, B. P. Weiss, P. F. Hoffman, Combined paleomagnetic, isotopic, and stratigraphic evidence for true polar wander from the Neoproterozoic Akademikerbreen Group, Svalbard, Norway. *GSA Bulletin* **118**, 1099–1124 (2006).

11. N. L. Swanson-Hysell, A. C. Maloof, D. J. Condon, G. R. T. Jenkin, M. Alene, M. M. Tremblay, T. Tesema, A. D. Rooney, B. Haileab, Stratigraphy and geochronology of the Tambien Group, Ethiopia: Evidence for globally synchronous carbon isotope change in the Neoproterozoic. *Geology* **43**, 323–326 (2015).

12. Y. Park, N. L. Swanson-Hysell, H. Xian, S. Zhang, D. J. Condon, H. Fu, F. A. Macdonald, A consistently high-latitude south China from 820 to 780 Ma: Implications for exclusion from Rodinia and the feasibility of large-scale true polar wander. *J. Geophys. Res. Solid Earth* **126**, e2020JB021541 (2021).

13. R. N. Mitchell, T. M. Kilian, T. D. Raub, D. A. Evans, W. Bleeker, A. C. Maloof, Sutton hotspot: Resolving Ediacaran-Cambrian Tectonics and true polar wander for Laurentia. *Am. J. Sci.* **311**, 651–663 (2011).

14. B. Robert, J. Besse, O. Blein, M. Greff-Lefftz, T. Baudin, F. Lopes, S. Meslouh, M. Belbadaoui, Constraints on the Ediacaran inertial interchange true polar wander hypothesis: A new paleomagnetic study in Morocco (West African craton). *Precambrian Res.* **295**, 90–116 (2017).

15. B. Robert, M. Greff-Lefftz, J. Besse, True polar wander: A key indicator for plate configuration and mantle convection during the late Neoproterozoic. *Geochem. Geophys. Geosyst.* **19**, 3478–3495 (2018).

16. V. Pavlov, Y. Gallet, Katav limestones: A unique example of remagnetization or an ideal recorder of the Neoproterozoic geomagnetic field? *Izv. Phys. Solid Earth* **45**, 31–40 (2009).

17. V. Pavlov, Y. Gallet, Variations in geomagnetic reversal frequency during the Earth's middle age. *Geochem. Geophys. Geosyst.* **11**, (2010).

18. A. Abrajevitch, R. Van der Voo, Incompatible Ediacaran paleomagnetic directions suggest an equatorial geomagnetic dipole hypothesis. *Earth Planet. Sci. Lett.* **293**, 164–170 (2010).

19. R. K. Bono, J. A. Tarduno, A stable Ediacaran Earth recorded by single silicate crystals of the ca. 565 Ma Sept-Îles intrusion. *Geology* **43**, 131–134 (2015).

20. D. A. D. Evans, True polar wander and supercontinents. *Tectonophysics* **362**, 303–320 (2003).

21. J.-C. Zhou, X.-L. Wang, J.-S. Qiu, Geochronology of Neoproterozoic mafic rocks and sandstones from northeastern Guizhou, South China: Coeval arc magmatism and sedimentation. *Precambrian Res.* **170**, 27–42 (2009).

22. J. Yao, P. A. Cawood, L. Shu, G. Zhao, Jiangnan Orogen, South China: A ~970–820 Ma Rodinia margin accretionary belt. *Earth Sci. Rev.* **196**, 102872 (2019).

23. P. L. McFadden, A new fold test for palaeomagnetic studies. *Geophys. J. Int.* **103**, 163–169 (1990).

24. G. S. Watson, R. J. Enkin, The fold test in paleomagnetism as a parameter estimation problem. *Geophys. Res. Lett.* **20**, 2135–2137 (1993).

25. T. Veikkolainen, L. J. Pesonen, Palaeosecular variation, field reversals and the stability of the geodynamo in the Precambrian. *Geophys. J. Int.* **199**, 1515–1526 (2014).

26. M. H. L. Deenen, C. G. Langereis, D. J. J. van Hinsbergen, A. J. Biggin, Geomagnetic secular variation and the statistics of palaeomagnetic directions. *Geophys. J. Int.* **186**, 509–520 (2011).

27. T. Veikkolainen, L. J. Pesonen, Precambrian geomagnetic field—An overview, in *Ancient Supercontinents and the Paleogeography of the Earth*, L. J. Pesonen, D. A. D. Evans, S.-A. Elming, J. M. Salminen, T. Veikkolainen, Eds. (Elsevier, 2021), pp. 81–108.

28. Z.-X. Li, D. A. D. Evans, S. Zhang, A 90° spin on Rodinia: Possible causal links between the Neoproterozoic supercontinent, superplume, true polar wander and low-latitude glaciation. *Earth Planet. Sci. Lett.* **220**, 409–421 (2004).

29. T.-T. Wang, S. Zhang, J. Ramezani, Age recalibration of the Xiaofeng Dykes, South China, and its implications for true polar wander at ~820 Ma. *Acta Geologica Sinica* **90**, 47–47 (2016).

30. D. J. J. van Hinsbergen, B. Steinberger, P. V. Doubrovine, R. Gassmöller, Acceleration and deceleration of India-Asia convergence since the Cretaceous: Roles of mantle plumes and continental collision. *J. Geophys. Res. Solid Earth* **116**, (2011).

31. S. Zahirovic, R. D. Müller, M. Seton, N. Flament, Tectonic speed limits from plate kinematic reconstructions. *Earth Planet. Sci. Lett.* **418**, 40–52 (2015).

32. H. Xian, S. Zhang, H. Li, T. Yang, H. Wu, Geochronological and palaeomagnetic investigation of the Madiyi Formation, lower Banxi Group, South China: Implications for Rodinia reconstruction. *Precambrian Res.* **336**, 105494 (2020).

33. L. Chang, S. Zhang, H. Li, H. Xian, H. Wu, T. Yang, New paleomagnetic insights into the neoproterozoic connection between South China and india and their position in rodinia. *Geophys. Res. Lett.* **49**, (2022).

34. P. A. Cawood, G. Zhao, J. Yao, W. Wang, Y. Xu, Y. Wang, Reconstructing south China in Phanerozoic and Precambrian supercontinents. *Earth Sci. Rev.* **186**, 173–194 (2018).

35. H. J. Walderhaug, T. H. Torsvik, E. A. Eide, B. Sundvoll, B. Bingen, Geochronology and palaeomagnetism of the Hunnendalen dykes, SW Norway: Implications for the Sveconorwegian apparent polar wander loop. *Earth Planet. Sci. Lett.* **169**, 71–83 (1999).

36. L. M. Fairchild, N. L. Swanson-Hysell, J. Ramezani, C. J. Sprain, S. A. Bowring, The end of Midcontinent Rift magmatism and the paleogeography of Laurentia. *Lithosphere* **9**, 117–133 (2017).

37. P. Y. J. Antonio, L. Baratoux, R. I. F. Trindade, S. Rousse, A. Ayite, C. Lana, M. Macouin, E. W. K. Adu, C. Sanchez, M. A. L. Silva, A. S. Firmin, West Africa in Rodinia: High quality paleomagnetic pole from the ~860 Ma Manso dyke swarm (Ghana). *Gondwana Res.* **94**, 28–43 (2021).

38. S. Zhong, N. Zhang, Z. X. Li, J. H. Roberts, Supercontinent cycles, true polar wander, and very long-wavelength mantle convection. *Earth Planet. Sci. Lett.* **261**, 551–564 (2007).

39. X. Le Pichon, M. Jellinek, A. Lenardic, A. M. C. Şengör, C. İmren, Pangea migration. *Tectonics* **40**, e2020TC006585 (2021).

40. J. W. Crowley, M. Gérault, R. J. O'Connell, On the relative influence of heat and water transport on planetary dynamics. *Earth Planet. Sci. Lett.* **310**, 380–388 (2011).

41. Z. X. Li, S. V. Bogdanova, A. S. Collins A. Davidson, B. De Waele, R. E. Ernst, I. C. W. Fitzsimons, R. A. Fuck, D. P. Gladkochub, J. Jacobs, K. E. Karlstrom, S. Lu, L. M. Natapov, V. Pease, S. A. Pisarevsky, K. Thrane, V. Vernikovsky, Assembly, configuration, and break-up history of Rodinia: A synthesis. *Precambrian Res.* **160**, 179–210 (2008).

42. H. Van Avendonk, J. Davis, J. Harding, Decrease in oceanic crustal thickness since the breakup of Pangaea. *Nat. Geosci.* **10**, 58–61 (2017).

43. P. A. Brandl, M. Regelous, C. Beier, K. M. Haase, High mantle temperatures following rifting caused by continental insulation. *Nat. Geosci.* **6**, 391–394 (2013).

44. M. Brown, C. L. Kirkland, T. E. Johnson, Evolution of geodynamics since the Archean: Significant change at the dawn of the Phanerozoic. *Geology* **48**, 488–492 (2020).

45. M. Tang, X. Chu, J. Hao, B. Shen, Orogenic quiescence in Earth's middle age. *Science* **371**, 728–731 (2021).

46. A. Lenardic, L. Moresi, A. M. Jellinek, C. J. O'Neill, C. M. Cooper, C. T. Lee, Continents, supercontinents, mantle thermal mixing, and mantle thermal isolation: Theory, numerical simulations, and laboratory experiments. *Geochem. Geophys. Geosyst.* **12**, Q10016 (2011).

47. A. M. Jellinek, A. Lenardic, R. T. Pierrehumbert, Ice, fire, or fizzle: The climate footprint of Earth's supercontinental cycles. *Geochem. Geophys. Geosyst.* **21**, (2020).

48. A. Lenardic, A supercontinental boost. *Nat. Geosci.* **10**, 4–5 (2017).

49. R. J. Stern, The evolution of plate tectonics. *Philos. Trans. R. Soc. A.* **376**, 20170406 (2018).

50. E. Sizova, T. Gerya, M. Brown, Contrasting styles of Phanerozoic and Precambrian continental collision. *Gondw. Res.* **25**, 522–545 (2014).

51. C. Herzberg, K. Condie, J. Korenaga, Thermal history of the Earth and its petrological expression. *Earth Planet. Sci. Lett.* **292**, 79–88 (2010).

52. P. A. Cawood, R. A. Strachan, S. A. Pisarevsky, D. P. Gladkochub, J. B. Murphy, Linking collisional and accretionary orogens during Rodinia assembly and breakup: Implications for models of supercontinent cycles. *Earth Planet. Sci. Lett.* **449**, 118–126 (2016).

53. R. N. Mitchell, T. M. Kilian, D. A. Evans, Supercontinent cycles and the calculation of absolute palaeolongitude in deep time. *Nature* **482**, 208–211 (2012).

54. Y. Gao, S. Zhang, H. Zhao, Q. Ren, T. Yang, H. Wu, H. Li, North China block underwent simultaneous true polar wander and tectonic convergence in late Jurassic: New paleomagnetic constraints. *Earth Planet. Sci. Lett.*, **567**, 117012 (2021).

55. A. S. Merdith, A. S. Collins, S. E. Williams, S. Pisarevsky, J. D. Foden, D. B. Archibald, M. L. Blades, B. L. Alessio, S. Armistead, D. Plavsa, C. Clark, R. D. Müller, A full-plate global reconstruction of the Neoproterozoic. *Gondw. Res.* **50**, 84–134 (2017).

56. N. L. Swanson-Hysell, J. Ramezani, L. M. Fairchild, I. R. Rose, Failed rifting and fast drifting: Mid-continent rift development, Laurentia’s rapid motion and the driver of Grenvillian orogenesis. *GSA Bulletin* **131**, 913–940 (2019).

57. D. J. Condon, B. Schoene, N. M. McLean, S. A. Bowring, R. R. Parrish, Metrology and traceability of U–Pb isotope dilution geochronology (EARTHTIME Tracer Calibration Part I). *Geochim. Cosmochim. Acta* **164**, 464–480 (2015).

58. J. F. Bowring, N. M. McLean, S. A. Bowring, Engineering cyber infrastructure for U–Pb geochronology: Tripoli and U–Pb_Redux. *Geochem. Geophys. Geosyst.* **12**, Q0AA19 (2011).

59. P. Vermeesch, IsoplotR: A free and open toolbox for geochronology. *Geosci. Front.* **9**, 1479–1493 (2018).

60. J. L. Kirschvink, The least-squares line and plane and the analysis of palaeomagnetic data. *Geophys. J. Roy. Astron. Soc.* **62**, 699–718 (1980).

61. R. Fisher, Dispersion on a sphere. *Proc. R. Soc. A* **217**, 295–305 (1953).

62. C. H. Jones, User-driven integrated software lives: "Paleomag" paleomagnetics analysis on the Macintosh. *Comput. Geosci.* **28**, 1145–1151 (2002).

63. L. Tauxe, R. Shaar, L. Jonestrask, N. L. Swanson-Hysell, R. Minnett, A. A. P. Koppers, C. G. Constable, N. Jarboe, K. Gaastra, L. Fairchild, PmagPy: Software package for paleomagnetic data analysis and a bridge to the Magnetics Information Consortium (MagIC) Database. *Geochem. Geophys. Geosyst.* **17**, 2450–2463 (2016).

64. J. Zhang, T. Ye, Y. Dai, J. Chen, H. Zhang, C. Dai, G. Yuan, K. Jiang, Provenance and tectonic setting transition as recorded in the Neoproterozoic strata, western Jiangnan Orogen: Implications for South China within Rodinia. *Geosci. Front.* **10**, 1823–1839 (2019).

65. J. Yao, L. Shu, P. A. Cawood, G. Zhao, Differentiating continental and oceanic arc systems and retro-arc basins in the Jiangnan orogenic belt, South China. *Geol. Mag.* **156**, 2001–2016 (2019).

66. G.-Y. Song, X.-Q. Wang, X.-Y. Shi, G.-Q. Jiang, New U-Pb age constraints on the upper Banxi Group and synchrony of the Sturtian glaciation in South China. *Geosci. Front.* **8**, 1161–1173 (2017).

67. Bureau of Geology and Mineral Resources of Guizhou Province, Regional Geology of Guizhou Province, Geological Publishing House, Beijing (in Chinese with English abstract) (1987).

68. J.-H. Zhao, M.-F. Zhou, D.-P. Yan, J.-P. Zheng; J.-W. Li, Reappraisal of the ages of Neoproterozoic strata in South China: No connection with the Grenvillian Orogeny. *Geology* **39**, 299–302 (2011).

69. M. Wang, C.-G. Dai, X.-H. Wang, H.-Z. Ma, C.-L. Peng, K.-D. Yang, Sedimentation age of the Fanjingshan Group in East Guizhou Province: Evidence from in-situ zircon LA-ICP-MS U-Pb dating. *Acta Petrol. Mineral.* **31**, 843–857 (in Chinese with English abstract) (2012).

70. L.-Z. Gao, J.-S. Chen, C.-G. Dai, X.-Z. Ding, X.-H. Wang, Y.-X. Liu, M. Wang, H. Zhang, SHRIMP zircon U-Pb dating of tuff in Fanjingshan Group and Xiajiang Group from Guizhou and Hunan Provinces and its stratigraphic implications. *Geol. Bull. China* **33**, 949–959 (in Chinese with English abstract) (2014).

71. C. Yan, L. Shu, M. Faure, Y. Chen, R. Huang, Time constraints on the closure of the Paleo–South China Ocean and the Neoproterozoic assembly of the Yangtze and Cathaysia blocks: Insight from new detrital zircon analyses. *Gondw. Res.* **73**, 175–189 (2019).

72. L. Tauxe, G. S. Watson, The fold test: An eigen analysis approach. *Earth Planet. Sci. Lett.* **122**, 331–341 (1994).

73. J. G. Meert, A. F. Pivarunas, D. A. D. Evans, S. A. Pisarevsky, L. J. Pesonen, Z. X. Li, S. Å Elming, S. R. Miller, S. Zhang, J. M. Salminen, The magnificent seven: A proposal for modest revision of the Van der Voo (1990) quality index. *Tectonophysics* **790**, 228549 (2020).

74. G. S. Watson, Large sample theory of the Langevin distribution, *J. Stat. Plann. Inference* **8**, 245–256 (1983).

75. R. R. Doell, Paleomagnetic secular variation study of lavas from the Massif central, France. *Earth Planet. Sci. Lett.* **8**, 352–362 (1970).

76. B. Efron, *The Jackknife, the Bootstrap and Other Resampling Plans* (Capital City Press, 1982).

77. X.-L. Wang, J.-C. Zhou, W.-L. Griffin, G. Zhao, J.-H. Yu, J.-S. Qiu, Y.-J. Zhang, G.-F. Xing, Geochemical zonation across a Neoproterozoic orogenic belt: Isotopic evidence from granitoids and metasedimentary rocks of the Jiangnan orogen, China. *Precambrian Res.* **242**, 154–171 (2014).

78. P. L. McFadden, M. W. McElhinny, The combined analysis of remagnetization circles and direct observations in palaeomagnetism. *Earth Planet. Sci. Lett.* **87**, 161–72 (1988).

79. D. F. Argus, R. S. Gross, An estimate of motion between the spin axis and the hotspots over the past century. *Geophys. Res. Lett.* **31**, L06614 (2004).

80. J. X. Mitrovica, J. Wahr, Ice Age Earth rotation. *Annu. Rev. Earth Planet. Sci.* **39**, 577–616 (2011).

81. N.-H Chan, J. X. Mitrovica, I. Matsuyama, K. Latychev, J. R. Creveling, S. Stanley, E. Morrow, The rotational stability of a convecting Earth: The Earth's figure and TPW over the last 100 Myr. *Geophys. J. Int.* **187**, 773–782 (2011).

82. B. Steinberger, M. L. Seidel, T. H. Torsvik, Limited true polar wander as evidence that Earth's nonhydrostatic shape is persistently triaxial. *Geophys. Res. Lett.* **44**, 827–834 (2017).

83. R. R. Fu, D. V. Kent, Anomalous Late Jurassic motion of the Pacific Plate with implications for true polar wander, *Earth Planet. Sci. Lett.* **490**, 20–30 (2018).

84. G. Muttoni, D. V. Kent, Jurassic monster polar shift confirmed by sequential paleopoles from Adria, promontory of Africa. *J. Geophys. Res. Solid Earth* **124**, 3288–3306 (2019).

85. R. R. Fu, D. V. Kent, S. R. Hemming, P. Gutiérrez, J. R. Creveling, Testing the occurrence of Late Jurassic true polar wander using the La Negra volcanics of northern Chile. *Earth Planet. Sci. Lett.* **529**, 115835 (2020).

86. R. Van der Voo, True polar wander during the middle Paleozoic?. *Earth Planet. Sci. Lett.* **122**, 239–243 (1994).

87. J. L. Kirschvink, R. L. Ripperdan, D. A. Evans, Evidence for a large-scale reorganization of Early Cambrian continental masses by inertial interchange true polar wander. *Science* **277**, 541–545 (1997).

88. R. N. Mitchell, D. A. D. Evans, T. M. Kilian, Rapid Early Cambrian rotation of Gondwana, *Geology* **38**, 755–758 (2010).

89. R. N. Mitchell, T. D. Raub, S. C. Silva, J. L. Kirschvink, Was the Cambrian explosion both an effect and an artifact of true polar wander? *Am. J. Sci.* **315**, 945–957 (2015).

90. R. K. Bono, J. A. Tarduno, F. Nimmo, R. D. Cottrell, Young inner core inferred from Ediacaran ultra-low geomagnetic field intensity. *Nat. Geosci.* **12**, 143–147 (2019).

91. V. V. Shcherbakova, V. G. Bakhmutov, D. Thallner, V. P. Shcherbakov, G. V. Zhidkov, A. J. Biggin, Ultra-low palaeointensities from East European Craton, Ukraine support a globally anomalous palaeomagnetic field in the Ediacaran. *Geophys. J. Int.* **220**, 1928–1946 (2020).

92. F. A. Macdonald, M. D. Schmitz, J. L. Crowley, C. F. Roots, D. S. Jones, A. C. Maloof, J. V. Strauss, P. A. Cohen, D. T. Johnston, D. P. Schrag, Calibrating the Cryogenian. *Science* **327**, 1241–1243 (2010).

93. Y. Park, N. L. Swanson-Hysell, S. A. MacLennan, A. C. Maloof, M. Gebreslassie, M. M. Tremblay, B. Schoene, M. Alene, E. S. C. Anttila, T. Tesema, B. Haileab, The lead-up to

the Sturtian Snowball Earth: Neoproterozoic chemostratigraphy time-calibrated by the Tambien Group of Ethiopia. *GSA Bulletin* **132**, 1119–1149 (2020).

94. X. Jing, Z. Yang, D. A. D. Evans, Y. Tong, Y. Xu, H. Wang, A pan-latitudinal Rodinia in the Tonian true polar wander frame. *Earth Planet. Sci. Lett.* **530**, 115880 (2019).

95. C. Maijer, R. H. Verschure, Petrology and isotope geology of the Hunnedalen monzonoritic dike swarm, SW Norway: The Hunnedalen dike swarm as a possible late expression of Egersund anorthosite magmatism. *NGU-Bull* **434**, 83–104 (1998).

96. I. M. Gorokhov, T. S. Zaitseva, A. B. Kuznetsov, G. V. Ovchinnikova, M. M. Arakelyants, V. P. Kovach, G. V. Konstantinova, T. L. Turchenko, I. M. Vasil'eva, Isotope systematics and age of authigenic minerals in shales of the upper riphean inzer formation, South Urals. *Stratigr. Geol. Correl.* **27**, 133–158 (2019).

97. H.-Q. Zhao, S.-H Zhang, J.-K. Ding, L.-X. Chang, Q. Ren, H.-Y. Li, T.-S. Yang, H.-C. Wu, New geochronologic and paleomagnetic results from early Neoproterozoic mafic sills and late Mesoproterozoic to early Neoproterozoic successions in the eastern North China Craton, and implications for the reconstruction of Rodinia. *GSA Bulletin* **132**, 739–766 (2020).

98. A. H. Jaffey, K. F. Flynn, L. E. Glendenin, W. C. Bentley, A. M. Essling, Precision measurement of half-lives and specific activities of ^{235}U and ^{238}U. *Phys. Rev. C* **4**, 1889–1906 (1971).

99. S. Zhang, H. Li, G. Jiang, D. A. D. Evans, J. Dong, H. Wu, T. Yang, P. Liu, Q. Xiao, New paleomagnetic results from the Ediacaran Doushantuo formation in South China and their paleogeographic implications. *Precambrian Res.* **259**, 130–142 (2015).

100. S. Zhang, D. A. D. Evans, H. Li, H. Wu, G. Jiang, J. Dong, Q. Zhao, T. D. Raub, T. Yang, Paleomagnetism of the late Cryogenian Nantuo Formation and paleogeographic implications for the South China Block. *J. Asian Earth Sci.* **72**, 164–177 (2013).

101. X. Jing, Z. Yang, Y. Tong, Z. Han, A revised paleomagnetic pole from the mid-Neoproterozoic Liantuo Formation in the Yangtze block and its paleogeographic implications. *Precambrian Res.* **268**, 194–211 (2015).

102. X. Jing, D. A. D. Evans, Z. Yang, Y. Tong, Y. Xu, H. Wang, Inverted south China: A novel configuration for Rodinia and its breakup. *Geology* **49**, 463–467 (2021).

103. J. Niu, Z.-X. Li, W. Zhu, Palaeomagnetism and geochronology of mid- Neoproterozoic Yanbian dykes, South China: Implications for a c. 820–800 Ma true polar wander event and the reconstruction of Rodinia. *Geol. Soc. Lond. Spec. Publ.* **424**, 191–211 (2016).

104. B.-C. Huang, Y.-X. Zhou, R.-X. Zhu, Discussions on Phanerozoic evolution and formation of continental China, based on paleomagnetic studies. *Earth Sci. Front.* **15**, 348–359 (2008).

105. R. J. Enkin, Z. Yang, Y. Chen, V. Courtillot, Paleomagnetic constraints on the geodynamic history of the major blocks of China from the Permian to the present. *J. Geophys. Res. Solid Earth* **97**, 13953–13989 (1992).

106. X. Zhao, R. S. Coe, S. A. Gilder, G. M. Frost, Palaeomagnetic constraints on the palaeogeography of China: Implications for Gondwanaland. *Aust. J. Earth Sci.* **43**, 643–672 (1996).

107. H.-N. Wu, R.-X. Zhu, L.-X. Bai, B. Guo, J.-J. Lü, Revised apparent polar wander path of the Yangtze Block and its tectonic implications. *Sci. China Ser. D Earth Sci.* **41**, 78–90 (1998).

108. S. Zhang, H. Zhu, X. Meng, New paleomagnetic results from the Devonian-Carboniferous successions in the Southern Yangtze Block and their paleogeographic implications. *Acta Geol. Sin.* **75**, 303–313 (2001).

109. H. Xian, S. Zhang, H. Li, Q. Xiao, L. Chang, T. Yang, H. Wu, How did South China connect to and separate from Gondwana? New paleomagnetic constraints from the Middle Devonian Red Beds in South China. *Geophys. Res. Lett.* **46**, 7371–7378 (2019).

110. N. D. Opdyke, K. Huang, G. Xu, W.-Y. Zhang, D. V. Kent, Paleomagnetic results from the Silurian of the Yangtze paraplatform. *Tectonophysics* **139**, 123–132 (1987).

111. K. Huang, N. D. Opdyke, R. X. Zhu, Further paleomagnetic results from the Silurian of the Yangtze Block and their implications. *Earth Planet. Sci. Lett.* **175**, 191–202 (2000).

112. Z. Yang, Z. Sun, T. Yang, J. Pei, A long connection (750–380 Ma) between South China and Australia: Paleomagnetic constraints. *Earth Planet. Sci. Lett.* **220**, 423–434 (2004).

113. J.-L Lin, M. Fuller, W.-Y. Zhang, Paleogeography of the North And South China blocks during the Cambrian. *J. Geodyn.* **2**, 91–114 (1985).

114. D. A. D. Evans, The palaeomagnetically viable, long-lived and all-inclusive Rodinia supercontinent reconstruction. *Geol. Soc. Lond. Spec. Publ.* **327**, 371–404. (2009).

115. D. A. D. Evans, R. V. Veselovsky, P. Y. Petrov, A. V. Shatsillo, V. E. Pavlov, Paleomagnetism of Mesoproterozoic margins of the Anabar Shield: A hypothesized billion-year partnership of Siberia and northern Laurentia. *Precambrian Res.* **281**, 639–655 (2016).

116. J. Ding, S. Zhang, D. A. D. Evans, T. Yang, H. Li, H. Wu, J. Chen, North China craton: The conjugate margin for northwestern Laurentia in Rodinia. *Geology* **49**, 773–778 (2021).

117. Z.-X. Li, D. A. D. Evans, Late Neoproterozoic 40° intraplate rotation within Australia allows for a tighter-fitting and longer-lasting Rodinia. *Geology* **39**, 39–42 (2011).
